# Nanoparticulate System for the Transdermal Delivery of Catechin as an Antihypercholesterol: In Vitro and In Vivo Evaluations

**DOI:** 10.3390/ph15091142

**Published:** 2022-09-13

**Authors:** Soraya Ratnawulan Mita, Marline Abdassah, Unang Supratman, Yoshihito Shiono, Driyanti Rahayu, Iyan Sopyan, Gofarana Wilar

**Affiliations:** 1 Department of Pharmaceutics and Pharmaceutical Technology, Faculty of Pharmacy, Universitas Padjadjaran, Jatinangor 45363, Indonesia; 2 Department of Chemistry, Faculty of Mathematics and Science, Universitas Padjadjaran, Jatinangor 45363, Indonesia; 3 Central Laboratory, Universitas Padjadjaran, Jatinangor 45363, Indonesia; 4 Department of Food, Life, and Environmental Sciences, Faculty of Agriculture, Yamagata University, Yamagata 997-8555, Japan; 5 Department of Analytical and Medicinal Chemistry, Faculty of Pharmacy, Universitas Padjadjaran, Jatinangor 45363, Indonesia; 6 Department of Pharmacology and Clinical Pharmacy, Faculty of Pharmacy, Universitas Padjadjaran, Jatinangor 45363, Indonesia

**Keywords:** catechin, transethosome, gel, antihypercholesterol

## Abstract

Gambir (*Uncaria gambir*, Roxb.) contains catechins that is often empirically used to treat various diseases. Catechins can reduce cholesterol levels by inhibiting coenzyme HMG-CoA reductase that plays a role in cholesterol metabolism. Research has been carried out covering the optimization of transethosomal catechins, the formulation of Transethosomal Catechin Gel (TCG) and Non-Transethosomal Catechin Gel (NTCG), which were then tested for catechin permeation from these gel preparations in vitro using Franz’s diffusion cell with PTFE membranes. The anti-hypercholesterol activity test was carried out with Simvastatin orally as a positive control using 25 male Wistar rats (*Rattus norvegicus*). The catechin transetosomes have a size of 176.1 ± 5.8 nm, Zeta potential −11.6 ± 5.28, and Entrapment Efficacy of 96.77% ± 0.05. The result of cumulative catechins that permeated from TCG and NTCG were and 172.454 ± 5.287 and 112.741 ± 2.241 μg respectively. Permeation test graphs showed similar permeation and flux profiles. TCG can reduce total cholesterol and LDL (Low Density Lipoprotein) values in rats by 39.77% and 51.52% respectively during 14 days of use.

## 1. Introduction

Hypercholesterolemia increases the risk of heart disease and stroke. Overall, one third of heart disease is caused by high cholesterol and is estimated to cause 2.6 million deaths (4.5% of the total). In 2008, the prevalence of hypercholesterolemia was highest in Europe (54% for both genders), followed by Americans (48% for both genders), meanwhile Africa and Southeast Asia show the lowest percentages (22.6% and 29.0%). In Indonesia, the prevalence of hypercholesterolemia is 9.3% (aged 25–34 years). This number increased by 15.5% in people aged 55–64 years. Hypercholesterolemia is more common in women (14.5%) than men (8.6%). Hypercholesterolemia is a leading cause of atherosclerosis [1].

Many classes of drugs are intended for the treatment of hypercholesterolemia, one of which is statins. Statins have become the drug of choice in reducing cholesterol, given the large decrease in cholesterol concentration which they can produce [2]. However, long-term use of statins does not rule out the possibility of unwanted effects. The most common effect is myopathy currently; antihypercholesterol drugs are in oral preparation form such as tablets or caplets. The novelty in this research is the creation of an antihypercholesterol drug that can be delivered transdermally [3]. Transdermal drugs are applied to the skin topically but can penetrate the skin barrier and enter the blood circulation which then has a systemic effect. This study combines liposomal and transdermal forms. Transdermal application has several advantages, including convenience and comfort when used by patients. This preparation can be an option for those who find it difficult to take medication orally [4]. Besides, there are long-term effects of statins that may occur such as bloating, dyspepsia, constipation, myalgia, rhabdomyolysis, insomnia and increased blood sugar [5].

One of the plants that has the potential in handling hypercholesterolemia is gambir (*Uncaria gambir*, Robx). This is because the content of catechin compounds in gambir can reduce Low Density Lipoprotein (LDL) cholesterol levels by increasing the activity of the lipoprotein lipase enzyme that plays a role in the metabolism of Very Low Density Lipoprotein (VLDL) [6]. Adelina and Arifayu’s research in 2018 supports the role of catechins in reducing LDL levels. In this study in silico, it was shown that catechins were able to increase LDL receptor expression [7].

Catechins belong to the polyphenol group of flavonoids that have high concentrations in vegetables and fruits. The main sources of catechins are gambir, grapes, apples, pears, cherries, and green tea. Although catechins are not essential nutrients for humans, the use of catechins can improve health in preventing various diseases [8]. Catechins are found in large quantities in the *Uncaria gambir* plant. According to the Indonesian Herbal Pharmacopoeia, the catechin content in gambir plants is at least 90% [9]. *Uncaria gambir* belongs to the Rubiaceae family and spread in Malaysia, Singapore, and Indonesia [10]. Gambir plants in Indonesia are found in West and South Sumatra, Java, and Kalimantan. Gambir (*Uncaria gambir* Roxb.) belongs to the family Rubiaceae, genus Uncaria. This plant is a climbing shrub with an elongated branching. It has short leaf stalks, oval-shaped leaves with tapered ends and a hairless leaf surface. Its flowers have short petals, funnel-shaped crown (like a coffee flower) with a pink or green colour, five stamens, and a two chambers capsule. The fruit shape is round like an egg with about 1.5 cm in length and has black colour [11,12].

Gambir is a native plant of Southeast Asia, particularly Indonesia and Malaysia [12]. According to Coordinating Ministry for Economic Affairs of the Republic of. Indonesia Data 2021, Indonesia contributes around 80% of gambir commodities in the world market, while West Sumatra supplies 80–90% of the total Indonesian production, and 90% of gambir production in West Sumatra comes from Fifty Cities District. There are four types of gambir: mancik riau, riau sieve, and cubadak [13]. Generally, gambir is cultivated on land with an altitude 650–800 m a.s.l. with a flat-to-topographical hillside. The gambir plant is usually found as cultivars of gambir reported in West Sumatera, Indonesia, namely shrimp gambir, a crop plantation in the yard or garden on the edge of the forest [12].

The leaves of gambir extract contains catechu tannat acid (tannin), catechin, pyrocatechol, fluorine, wax, and oil. The main components of gambir are Catechu Tannic acid (20–50%), catechins (7–73%), and pure pyrocatechol that are slightly soluble in cold water but soluble in hot water, alcohol and ethyl acetate [14]. Indonesian Herbal Pharmacopoeia 2017 states gambir contains tannin levels of not less than 90% calculated as catechins.

Classification of Plants

Kingdom: PlantaeDivision: MagnoliophytaClass: MangnoliopsidaSub-class: AsteridaeOrder: RubialesFamily: RubiaceaeGenus: UncariaSpecies: *Uncaria gambir* (Hunter) Roxb.Synonyms: *Uncaria gambir* Roxb. *Uncaria acida* Roxb. *Nauclea gambir* Hunt. *Ourouparia gambir* Baill [14].

The catechins contain two aromatic rings and several hydroxyl groups, based on which they are categorized into two groups: free catechins and esterified catechins [15]. Many studies on catechin compounds state that it has cholesterol-lowering activity by inhibiting the HMG Co A reductase enzyme, like in a statin group. This enzyme plays a role in the biosynthesis of cholesterol in the body [16,17]. Statins are a group of compounds commonly used to treat hypercholesterolemia. The drawback of catechins is their low bioavailability value, which is <5% [18].

Transethosomes are a liposomal form which is a combination of ethosomes and transfersomes that was introduced by Song, 2012 [19]. The transethosomal formula contains ethanol, surfactants, phospholipids, and water [20]. Permeation of active compounds by transethosomes was better than the other Transethosomal preparations intended for transdermal delivery. Transdermal preparations are used as an alternative for drug delivery where the active compounds had considerable metabolism in the gastrointestinal tract. The transetosomal form can penetrate better than the ordinary liposome form because it is deformable; besides that, the alcohol in the formula makes the stratum corneum layer more permeable [21,22,23,24].

Liu et al., in 2018 investigated that flexseed oil, olive oil, and corn oil were able to increase the accessibility of axanthin encapsulated in oil-in-water nanoemulsions. There are also other studies that say curcumin made in the form of nanogels has anticancer properties that have been studied in three types of human cancer cells: breast cancer, hepatocellular carcinoma, and gastric cancer [25]. Tomato extract containing curcumin and lycopene as antioxidants can be encapsulated as nanoemulsions and as neutraceuticals [26]. Other studies mention the polyphenol content extracted from the leaves of the *Camellia sinensis* plant as anticancer and anti-inflammatory in the form of nanoemulsion preparations [27]. Red wine extract has the potential to make liposomes associated with polymers and have antioxidant activity [28]. Nanoparticles from ginkgo (melinjo) extract can increase the release of acetylcholine from the cerebral cortical synapses [29]. Thymoquinone, the bioactive component of Nigella sativa, when encapsulated in a lipid nanocarrier has six times higher bioavailability compared to free thymoquinone and increases gastrointestinal protective activity [30].

The application of nanotechnology in nutraceuticals has been studied intensively in recent years. Nanotechnology can be applied to nutraceutical delivery systems with the aim of increasing bioavailability so as to increase health benefits. The advantages of using nanotechnology for nutraceuticals are efficient encapsulation and good delivery and release [31]. The nanostructured lipid carrier (NLC) method has been investigated to be able to protect the phenol component extract obtained from the Hibiscus sabdariffa plant, where the plant has an effect on chronic diseases such as diabetes, hypertension, and obesity [32].

In this study, the formulation of transethosomal preparations from purified gambir extract was carried out using a two-level factorial design method. Factorial design is a method for determining the simultaneous influence of several factors and their interactions. In the two-level factorial design, there is a maximum level and a minimum level to determine the optimum number of variables used. The factorial design method was carried out to save costs and time efficiency in determining the optimal formula for transethosome, because it can determine effects and interactions systematically by only conducting a single study [33]. The optimum formula was obtained by analyzing the character of the transethosome so as to produce a good formula characterization such as particle size, polydispersity index, zeta potential, entrapment efficiency and particle morphology. Optimal transethosomes were isolated inside gel dosage forms that evaluated within in vitro and in vivo.

The novelty in this research is to make a transethosomal gel formula loaded with purified catechins with the aim of being an anticholesterol. So far, the dosage form of the drug as an anticholesterol is in the form of an oral dosage form, such as tablets or capsules.

## 2. Results and Discussion

### 2.1. Catecin Purification

#### 2.1.1. Characterization of Purified Catechin

The results of measuring the catechin concentration from the purification of gambir extract using HPLC showed that the catechin had a *R*_f_ of 2.534 which was similar to the catechin standard of 2.539. Based on the chromatogram, it can be concluded that the purification of gambir extract contains catechin compounds with a concentration that meets the requirements for use as a natural pharmaceutical preparation, which is above 90%.

#### 2.1.2. Phytochemial Study of *Uncaria gambir*

Gambir is included in the Rubiaceae family which is known have pharmacological activity. Catechins are the mayor compound in *Uncaria gambir*. In the Indonesian Herbal Pharmacopoeia was mentioned levels of catechins in the extract gambir amounted minimum of 90% [9]. Catecin as a major compound in *Uncaria gambir*. Catecin was isolated from the ethyl acetate and its structure was identified based on spectroscopic evidence. The structure of Catechin was confirmed through comparison of its spectroscopic datas of FTIR, MS, and NMR in Supplementary Materials (Figures S1–S8).

### 2.2. Catechin

**Figure 1:**
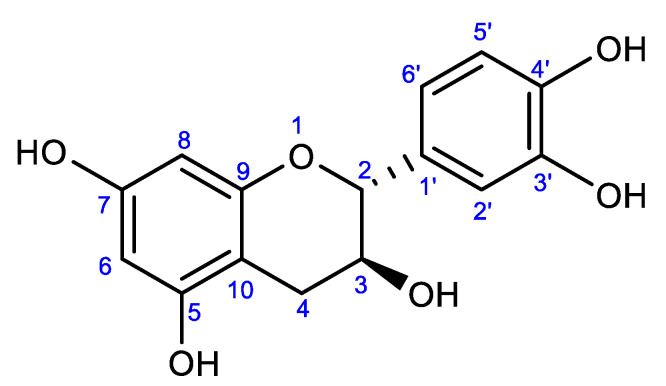
Molecular structure of catechin.

Catechins, also known as catechoic acid with chemical formula C_15_H_14_O_6_, are colorless, in their pure state they are very soluble in hot water but slightly insoluble in cold water, almost insoluble in chloroform, benzene and ether, and soluble in ethyl acetate and alcohol. It is weakly acidic (pKa 1 = 7.72 and pKa 2 = 10.22), stable at acidic pH (pH 2.8 and 4.9), easily oxidized at a pH close to neutral. The molecular structure of catechin is shown at Figure 1. Catechins are less stable to light and heat, and unstable if stored too long [34,35]. Their stability depends on the concentration of the phenolic components. Catechins will be more stable if they have a higher concentration of phenolic components.

High concentration of catechins from gambir extract obtained by repeated extraction was carried out along the principle of differences in the solubility properties of catechins and tannins in water. Repeated extraction was carried out using a solution of ethyl acetate or ethanol, followed by purification using amberlite absorbent [36].

### 2.3. Analysis on the Factorial Design

Formula optimization on transethosomes was carried out with the aim of estimating the optimum formula based on the resulting response variables to serve as a reference for the formulation of the base for the active substance to be used. The formula optimization method used is the Response Surface Method (RSM). RSM is a collection of statistical and mathematical techniques that are useful for developing, improving, and optimizing processes in which the response is influenced by several factors (independent variables). In addition to generating mathematical models, SRM can also explain the effect of independent variables, which explain a process. The main idea of this method is to determine the effect of the independent variable on the response, to obtain a model of the relationship between the independent variable and the response, and to obtain the process conditions that produce the best response. The advantages of the RSM method include that it does not require large amounts of experimental data and does not take a long time.

The experimental design used in this study was a two-level factorial design. The two-level factorial design method can estimate the factors that have the most influence on the specified response value and the interaction between factors on the determined response value. In addition to studying the interaction between factors, the two-level factorial design provides less data than the three-level factorial design so that it can save time and materials for use in research [33]. In this study, the factors used were phospholipids and tween 80, with the response variables in the form of particle size and polydispersity index, as it showed at Table 1. Particle size and polydispersity index values of the resulting transethosomes are influenced by variations in phospholipid concentrations and tween 80 (Table 2). Phospholipids serve as the main constituent components of vesicles. Phospholipids used in the form of Phospholipon, 90 G which contains phosphatidylcholine soybean granules of 94–102%.

Data processing using a two-level factorial design method with software Design Expert^®^ 11 (trial version) was carried out to find the formula that produced the most optimum response value. The response values tested in this software are particle size and polydispersity index. The response value that is entered into this factorial design will later determine which factors have the most influence on the intended response value, as well as study the interactions between factors that affect the response value.

Analysis of variation was performed on each parameter of particle size (PSA) and polydispersity index (PDI). This factorial design can provide a significance value for each test parameter on the response results in each formula with ANOVA. *p*-value less than 0.05 indicates that these factors show a significant difference in effect on the response to each formula. This shows that the different formulas for phospholipids and tween 80 affect the values for PSA and PDI.

The results of the analysis of response data on particle size and the polydispersity index on design expert 11 software (trial version) resulted in a general equation of factorial design of the relationship between factors and responses according to the general equation Y = b0 + baXA + bbXB + babXAXb as follows:

Final Equation in Terms of Actual Factors:

$$\begin{array}{ll} \mathrm{Particle}\;\mathrm{Size}= & -771.67+375.267\times \mathrm{Phospolipids}+213.67\times \mathrm{Tween}80\\ & -99.867\times \mathrm{Phospolipids}\times \mathrm{Tween}80 \end{array}$$

$$\begin{array}{ll} \mathrm{Polydispersity}\;\mathrm{Index} & \\ & =0.332083+0.0483167\times \mathrm{Phospolipids}-0.216167\times \mathrm{Tween}80\\ & +0.03850\times \mathrm{Phospolipids}\times \mathrm{Tween}80 \end{array}$$

Data analysis performed on Design Expert^®^ 11 provides recommendations for formulas with the best phospholipid and tween 80 compositions, and can predict the value of each particle size parameter and polydispersity index that will be generated from the formula. This software will present the response value of particle size and polydispersity index for each formula in the form of a 3-dimensional response surface graph, contour plot and regression equation model. A contour plot is a graph that shows a 3-dimensional surface of a response from its 2-dimensional shape (contour plot). The 3-dimensional response surface graph and the contour graph depict the relationship between particle size response and polydiversities index to the concentration of phospholipids and tween 80 in each transethosomal formula. The graph of the contour plot of the response has several colors. This color gradient indicates the significance of the desired response starting from dark blue to red. The blue area shows the lowest percent response value, while the red area shows the highest response value.

The three-dimensional graph is a visualization of the general polynomial equation that helps in determining the optimum point of each variable used. This polynomial equation is only valid and suitable for predicting particle size. The three-dimensional graph is a visualization of the general polynomial equation that helps in determining the optimum point of each variable used. This polynomial equation is only valid for predicting particle size in the range of phospholipid concentrations of 3–5% *w*/*w* and tween 80 1.5–2.5% *w*/*w*. The color difference on the curve indicates a change in the response value to the particle size, due to changes in the combination of variables. The difference in height on the surface shows different response values for each combination of formulas. The blue color indicates the lowest particle size response value of 128.8 nm, while the yellow color indicates the highest particle size response value of 811.7 nm. The highest particle size was found in formula 3 with a phospholipid concentration of 5% *w*/*w* and tween 80 was 1.5% *w*/*w*, while the lowest particle size response was found in formula 1 with a phospholipid concentration of 3% *w*/*w* and tween 80 of 2.5% *w*/*w*.

This particle size response contour plot maps phospholipids on the 1× axis, tween 80 on the ×2 axis, and the particle size response value as a contour. The size response graph shows that the particle size value will increase if the concentration of phospholipids and tween 80 used approaches the red or orange colored area, because in the red region the particle value produced is the highest. On the *x*-axis, it can be seen that the higher the concentration of phospholipids contained in the formula, the larger the particle size that will be produced. In contrast to the *y*-axis, the greater the amount of tween 80 concentration contained in the formula, the smaller the particle size produced. The formula recommended by the Design Expert^®^11 software will be predicted to have a particle size value of 129.8 nm (<200nm).

In the range of phospholipid concentrations of 3–5% *w*/*w* and tween 80 1.5–2.5% *w*/*w*, the color difference on the curve indicates a change in the response value to the particle size. The difference in height on the surface shows different response values for each combination of formulas. The blue color indicates the lowest particle size response value of 128.8 nm, while the yellow color indicates the highest particle size response value of 811.7 nm. The highest particle size was found in formula 3 with a phospholipid concentration of 3% *w*/*w* and tween 80 was 2.5% *w*/*w*, while the lowest particle size response was found in formula 1 with a phospholipid concentration of 3% *w*/*w* and tween 80 of 1.5% *w*/*w*.

The most recommended formula by the response surface method has a composition of 3% *w*/*w* phospholipid concentration and 2.5% *w*/*w* tween 80 concentration. The formula is predicted to have a particle size of 139.3 nm and a polydispersity index of 0.226. The range of values that meet the requirements for particle size is less than 300 nm and polydispersity index is less than 0.5 [37].

From the 3-dimensional response surface graph of the polydispersity index response, the equation model that describes the relationship between the polydispersity index and the concentration of phospholipids and tween 80 is a quadratic equation model that has a minimum polydispersity index value. The green color indicates the lowest polydispersity index response value, which is 0.019, while the red color indicates the highest polydispersity index response value, which is 0.594. The highest polydispersity index was found in formula 3, with a phospholipid concentration of 5% *w*/*w* and tween 80 of 1.5% *w*/*w*. The lowest polydispersity index response was also found in formula 2, with a phospholipid concentration of 3% *w*/*w* and tween 80 of 2.5% *w*/*w*.

**Figure 2:**
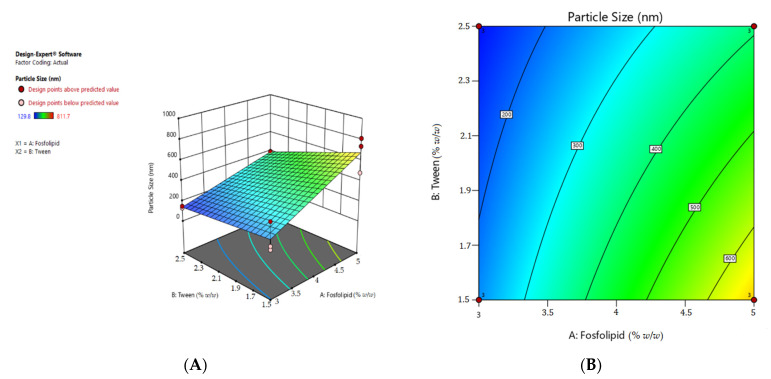

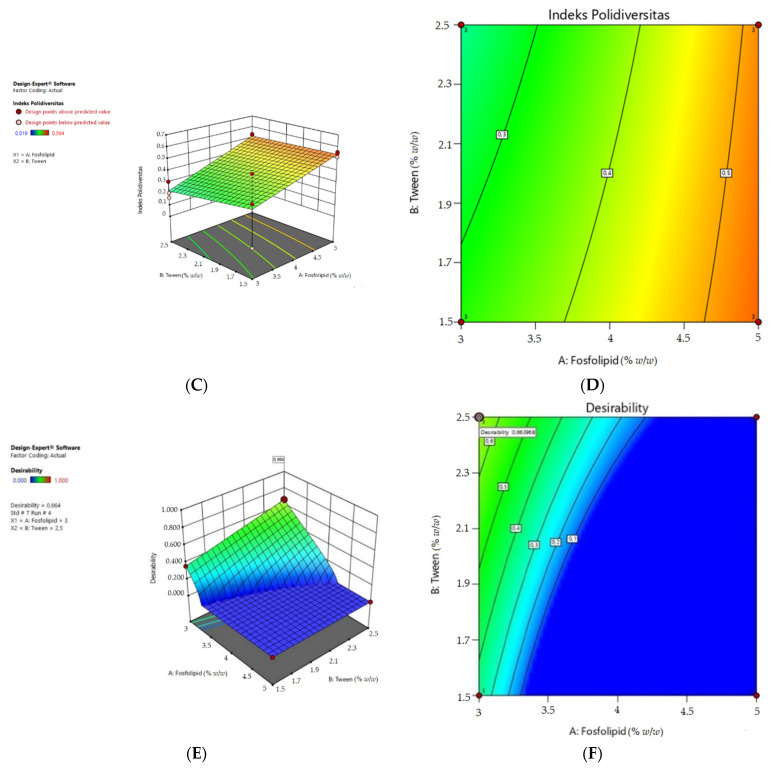
(**A**) 3D graphics of particle size response, (**B**) contour plot of particle size response, (**C**) 3D graphics of polydispersity index, (**D**) contour plot of polydispersity index, (**E**) 3D graphics of desirability, (**F**) contour plot of desirability.

This polydispersity index response graph depicts phospholipids on the *x*-axis, tween 80 on the *y*-axis, and the response value of the polydispersity index as a contour. The size response chart shows that the value of the polydispersity index will increase if the concentration of phospholipids and tween 80 used approaches the red area, because in the red area the polydispersity index value is the highest. On the *x*-axis, it can be seen that the higher the phospholipid in the formula, the higher the polydispersity index. In contrast to the *y*-axis, the greater the volume of tween 80, the smaller the polydispersity index. The formula recommended by the Design Expert^®^11 software will be predicted to have a polydispersity index value of 0.019 as the lowest polydispersity index response that showed at Figure 2. The polydispersity index is a condition in the distribution of particles that describes the level of uniformity of particle size in a component. The smaller the polydispersity index value, the more likely it is that the distribution of particles in a component has a good level of size uniformity [38].

Design Expert^®^ 11 provides the best formula solution based on the desirability value. The desirability value is the value of the optimization objective function, which indicates the program’s ability to fulfill the requirements based on the criteria set in the final product. The desirability value explains the close relationship, or not, a response value generated by the factor with the requirement value/ideal value. The desirability value has a range from 0–1. Where, the closer the desirability value is to a value of 1, the better the software’s ability to produce optimum formulas and meet the requirements. The actual purpose of optimization is not to be able to get a desirability value of 1, but to find the best conditions that consider all functions, factors, and responses.

In the criteria for the optimum acceptance of the transethosomal formula, the particle size value entered into the goal is the particle size value according to the requirements of the transethosomal preparation, which is less than 300 in the minimized category. In determining the optimum particle size value in the minimized category, the lower limit value of 100 is the lowest desired value and the upper limit value of 300 is the highest value that is still acceptable [21]. If the particle size value is getting closer to 100, the closer the particle size value to the optimal result will be, which will affect the desirability value. Determination of the value of the requirement for the polydispersity index used the manimize category; because the requirement for the value is less than 0.5, if the value of the polydispersity index is closer to 0, the transethosomal preparation is more homogeneous and has higher physical stability.

After determining the requirements for the optimum particle size and polydispersity index in transethosomal preparations, Design Expert^®^ 11 provides the most optimum composition of phospholipid and tween 80 formulas based on the highest desirability value. The best value of particle size, polydispersity index and desirability are in the Table 3.

Desirability indicates the possibility of obtaining a formula according to the desired criteria. Desirability is the optimization target value that can be achieved with a value range of 0–1. In the optimization of this formulation, the aim is to achieve the maximum desirability value by finding the best conditions that bring together all the test variables. The formula with the highest level of desirability will occupy the top position from the various suggestions offered. The higher the level of desirability, the closer the response results will be to the predicted value.

The Table 3 provides four recommendations for the best formula for the composition of phospholipids and tween 80, which produce the most optimum polydispersity index and particle size.

This contour plot depicts the phospholipid factor on the *x*-axis, tween 80 on the *y*-axis, and the desirability value as a contour. The desirability value has a range of 0 to 1. If the response is closer to the blue contour, then the resulting response does not meet the satisfaction of the particle size criteria and the optimum polydispersity index because the desirability value is close to 0. If the response is getting closer to the red contour, the resulting response will be increasingly fulfilled by the satisfaction of the particle size criteria, and the optimum polydispersity index because it is close to the desirability value of 1.

The most recommended formula by the response surface method has a composition of 3% *w*/*w* phospholipid concentration and 2.5% *w*/*w* tween 80 concentration. The formula is predicted to have a particle size of 139.3 nm and a polydispersity index of 0.226. Based on the optimization results, the formula best meets the desired optimization target because it has the highest desirability value of 0.664 (selected row). Desirability of 0.664 means that this formula will produce a product that has characteristics that match the optimization target of 66.4%. Accepted desirability value must be higher than 0.6.

### 2.4. Optimization of Transethosome Loaded with Catechin

After the optimal formula on the basis of transethosomes, namely formula 2 with a phospholipid composition of 3% *w*/*w* and tween 80 2.5% *w*/*w*, a formulation for transethosomal catechins was carried out with various characterizations to obtain catechin transethosomes. This has the appropriate characteristics. The optimized variable is the concentration of catechins, while the characterization or response to be carried out is particle size, polydispersity index, zeta potential, and entrapment efficiency.

The catechins used in this formula are the result of purification of gambir extract with *n*-hexane, ethyl acetate, and water as solvents; the catechin concentration obtained is 91.66% with characteristics that are in accordance with the requirements of the Indonesian Herbal Pharmacopoeia.

**Figure 3:**
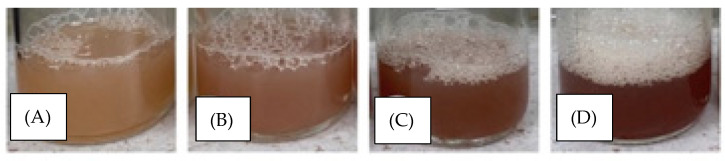
The result of the preparation of four catechin transethosomal formulas: (**A**) catechin concentration 0.5%; (**B**) 0.75% catechin concentration; (**C**) 1% catechin concentration; (**D**) catechin concentration 1.25%.

Formulation for transethosomal catechins was carried out with various characterizations to obtain catechin transethosomes, which has the appropriate characteristics. The visualization of transethosomal catechin showed in Figure 3. The optimized variable is the concentration of catechins, while the characterizations or responses to be carried out are particle size, polydispersity index, zeta potential, and entrapment efficiency (Table 4).

#### 2.4.1. Effect of Formulation Variable on the Thansethosome Properties

##### Particle Size Distribution

The result of characterization on particle size shows a value within the expected range for each transethosomal catechin formula that has been tested, which is still in the range of 100–300 nm [21]. The rank order of the mean particle size values of the catechin transethosomes was F1 < F2 < F3 < F4. Formula F1, with the lowest concentration of catechins, has the smallest mean particle size. In the results of the analysis, it was one way ANOVA found that the significance of the particle size was 0.001. This means that the particle size value between the formulas is significant, or H0 is rejected and H1 is accepted. In this test, the significance of the catechin concentration 0.5 to 0.75% was 0.571, which means the difference is not significant. For a concentration of 0.75 to 1%, a significance of 0.198 was obtained, which is not significant and for a concentration of 1 to 1.25%, a significance of 0.003 was obtained, which means significant. With this significance, it can be said that between the formulas with catechin concentrations of 0.5%, 0.75% and 1% there was no significant difference in particle size, but there was a significant difference at the concentration of 1.25% against other concentrations.

##### Polydispersity Index

In the polydispersity index test, the results that meet the acceptance criteria for each transethosomal catechin formula are 0–0.5. The polydispersity index of 0–0.5 indicates that the particle size distribution is narrow, which means it has high homogeneity, while the polydispersity index value of more than 0.5 indicates that the wide particle size distribution is low homogeneity. The value of the polydispersity index, which is closer to 0, is that it has a better particle size distribution [39]. Based on the criteria for the poly-dispersity index, the four formulations of transethosomal catechins met the standard. In the results of the analysis, it was one way ANOVA found that the significance of the particle size was 0.125. This means that the polydispersity index value between formulas is not significant, or H0 is accepted and H1 is rejected. The best polydispersity index which is closest to 0 is found in the formula with a catechin concentration of 1% with an average polydispersity index of 0.11. This means that the 1% catechin transethosomal formula is more homogeneous and has a more even distribution of particles compared to other formulas.

##### Zeta Potential

Zeta potential is a physical parameter used to predict vesicle stability [40]. All catechin transethosomal formulations showed negative zeta potential values as expected. This is due to the presence of ethanol, which gives a negative charge to the polar head groups of the phospholipids which creates an electrostatic repulsion. Negatively charged vesicles have better skin permeation properties than positively charged ones. However, the zeta potential value obtained does not match the criteria, which is greater than +30 mV or less than -30 mV. In this study, the surfactant used was tween 80, which is a non-ionic surfactant so that it does not give a charge to the particles. The zeta potential value is not suitable, because the tween 80 mechanism in increasing stability to create a barrier between the existing particles so that the particles do not combine and experience aggregation.

Zeta potential obtained is smaller than ±30 mV which causes the nanoparticle system to be less stable; however, in the presence of a non-ionic surfactant in the form of tween 80 can stabilize the solution with steric conditions at tween 80 [41]. A greater charge of the nanoparticles would be toxic to cells because it would damage cell membranes. Nanoparticles must have guaranteed stability by having sufficient charge and not being toxic to cells. According to previous studies, the potential zeta value in nanoparticle preparations below +30 mV can provide good value for biological activity or pharmacological effects. Based on this, the zeta potential cannot be used as a critical parameter and cannot be used as a determinant in choosing the optimum formula for catechin transethosomes.

##### Entrapment Efficiency

**Figure 4:**
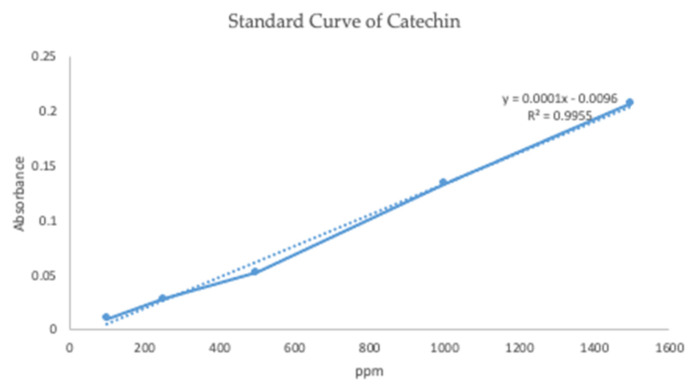
Standard curve of catechin.

Based on the standard curve measurement results, we obtained a regression equation that will be used to determine the level of entrapment efficiency of catechins in transethosomal preparations, namely *y* = 0.0001*x* − 0.0096 with a correlation coefficient (r) of 0.9955. The standard curve which is linear and in accordance with the requirements has a linearity value of r 0.99 (Figure 4). The value of r that meets the requirements can be interpreted that changes in levels will affect the absorbance value linearly.

In the results of the Kruskal–Wallis analysis, it was found that the significance of the particle size of 0.014 showed a significant or significant difference if the significance value of each treatment group was less than 0.05 (≤0.05). It can be said that H0 is rejected and H1 is accepted. The highest percentage of entrapment was owned by formula 4 with 1.25% catechins. However, between formulas 3 and 4 there is no significant difference so that formula 3 can be chosen as the optimum formula by considering the particle size. Furthermore, the formula use for the next step was formula contain 1% of catechin.

### 2.5. Characterization of Trannsethosomal Catechin 1%

In this research, the formulation of transethosomal preparations from purified gambir extract was carried out using a two-stage factorial design method. Factorial design included a method for determining the simultaneous effect of several factors and their interactions. In the two-level factorial design, there is a maximum level and a minimum level to determine the optimum number of variables used. The factorial design method was used to save costs and generate time efficiency in determining the optimal formula for transethosomes because it can determine effects and interactions systematically by only conducting a single study. The optimum formula was obtained by analyzing the character of the transethosomes so as to produce a good formula characterization such as particle size, polydispersity index, zeta potential and entrapment efficiency (Supplementary Materials, Figure S9).

#### 2.5.1. Morphological Characterization of the Transethosomes

**Figure 5:**
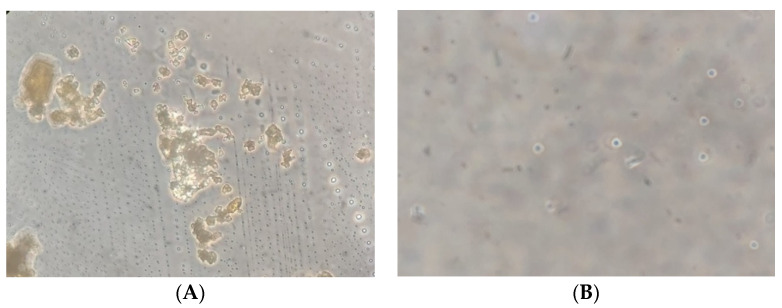

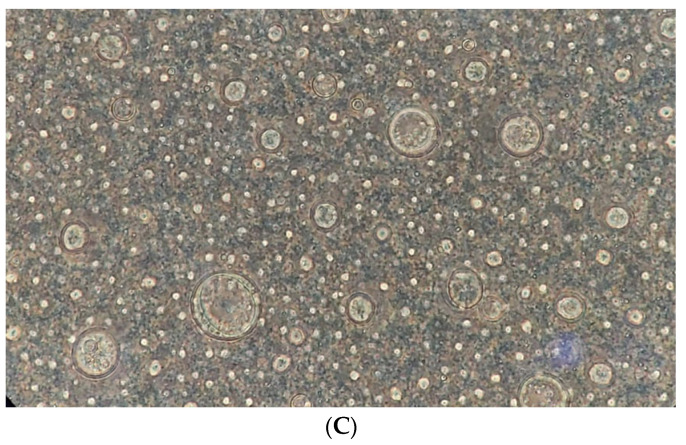
Micrograph of (**A**) purified catechin, (**B**) empty transethosome (**C**) transethosomal catechin in 400× magnification.

**Figure 6:**
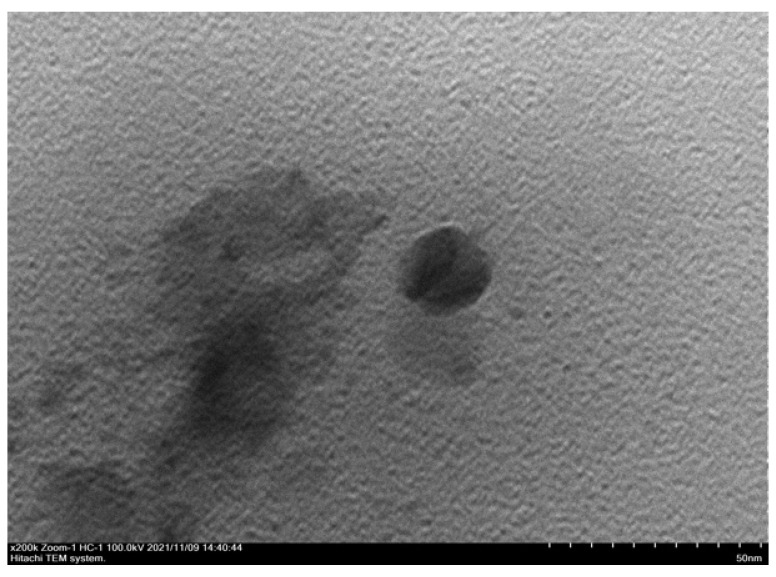
Morphology of transethosomal catechin using TEM.

According to the Figure 5 you can see a clear visual difference between the empty transethosomes and transethosomes that have been added with catechins. In the empty transethosomes, the core is seen in the form of small black dots indicating the water, buffer, and tween 80 that trapped in the transethosomes [42]. In the catechin transethosomes, the transethosomal nucleus looks more faded in color and some appear to be small spheres. The micrographs catechin transethosomes (Figure 5C) show that there are spheres of various sizes that have layers on the sides of the circumference. On the inside of the sphere, it is seen that the core mass of the transetosomes is formed. The morphology of the transethosome was characterized too by using a transmission electron microscope (TEM). The transthosomes showed as black dots in well-identified spherical shapes and homogenous and non-aggregated vesicles (Figure 6).

#### 2.5.2. Anticholesterol Assay

**Figure 7:**
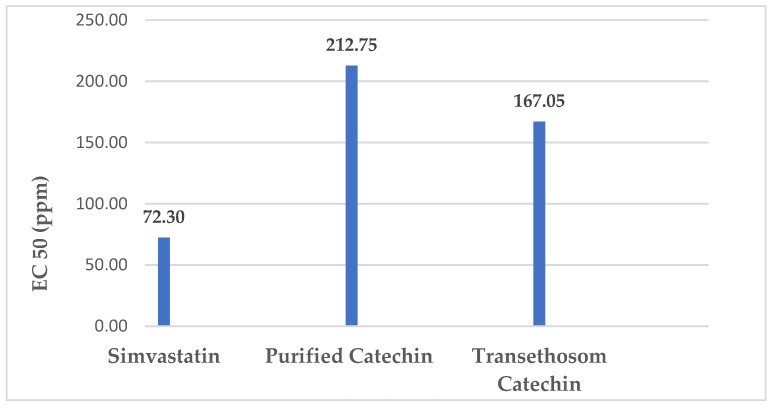
In vitro study of anticholesterolemic of cathechin performed to determine EC50 of Purified Catechin, Transethosom Catechin compared to Simvastatin. Transethoson cathechin formula enhanced the EC50 close to simvastatin as reference drug more effective than purified catechin.

The results of in vitro anticholesterol measurements showed that simvastatin as a positive control gave the best EC_50_ value. However, there are interesting data that show catechins that have been formulated into transethosomal forms to be giving better results as an anticholesterol versus purified catechins. The liposomal form can increase the EC_50_ value of anticholesterol because it can penetrate the gap between cells, which causes catechins to be delivered in greater quantities assisted by purified catechins. The particle size of catechins in the form of transethosomes is much smaller than that of purified catechin powder. This causes the catechins to be more soluble in the reagents used. The greater the amount of dissolved catechins, the more the number of catechins that react with cholesterol. The remaining free cholesterol that is measured in the solution becomes less. In addition, the presence of surfactants in the transethosomal formula was able to reduce the surface tension and free energy of catechin compounds. This increases the bioavailability of catechins. The liposomal form can increase the EC_50_ value of anticholesterol because it can penetrate the gap between cells, which causes catechins to be delivered in greater quantities assisted by purified catechins. The EC_50_ value of transethosomal catechins was 167.05 ppm, while the purified catechins was 212.75 ppm. Simvastatin as a positive control had an EC_50_ of 72.30 ppm (Figure 7). Based on this, the next step is to make a gel that loads transethosomes catechins.

### 2.6. Formulation of NTC and TC Gel

**Figure 8:**
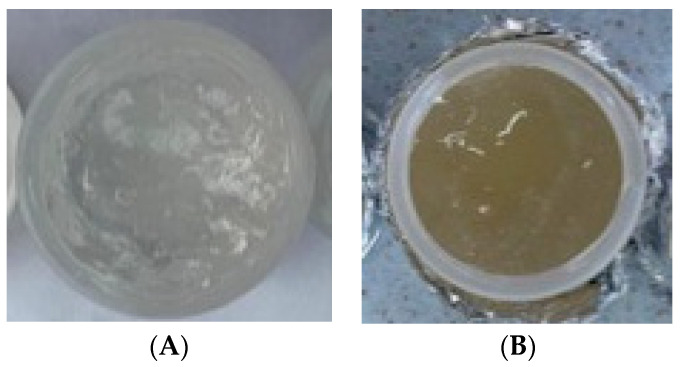
(**A**) NTC gel preparation; (**B**) TC gel preparation.

The non-transethosomal gel is in the form of a thick gel, has a characteristic odor, is clear, and is slightly yellowish in color (Figure 8). Meanwhile, the transethosomal gel is in the form of a thick gel, has a characteristic odor, is bright yellow-gray in color according to the standard Pantone card 453 C, and is slightly cloudy due to the presence of phospholipids.

The pH measurement was carried out in triples, with the average pH of the non-transethosomal gels being 5.90 ± 0.03 and the pH of transethosomal gels being 5.67 + 0.01. The pH of the preparation is in accordance with the pH range of the skin so that it fits the ideal requirements of transdermal preparations [43]. The measured viscosity of the non-transethosomal gel was 640.800 cPs and the transethosomal gel was 212.466.667 cPs.

### 2.7. In Vitro Permeation of Catechin from the Transethosomal Gel

During the penetration test with the Franz diffusion cell, the receptor compartment of the device was kept at a temperature of 32 + 0.5 °C, which represents body temperature. The receptor compartment fluid was stirred with a magnetic stirrer at a speed of 600 rpm to simulate body conditions and ensure homogeneity. This velocity is used because it is considered a suitable velocity to homogenize the fluid in the receptor compartment. At speeds lower than 600 rpm it will be difficult to homogenize the fluid in the receptor compartment [44].

In permease testing, drug levels will move from the donor compartment (high catechin concentration) to the receptor compartment which contains phosphate buffer medium pH 5.8. The receptor compartment has a large volume compared to the amount of substance that penetrates. This large volume aims to accommodate a large volume of liquid so as to create a sink condition. The sink condition is a condition where the volume of liquid to dissolve the substance is so large that it will not hinder drug penetration. This is because the large volume of fluid will not cause saturation in the receptor compartment. Drug penetration from the donor compartment to the receptor compartment is based on the principle of passive diffusion which uses the drug concentration gradient as the driving force for drug penetration.

Catechins that have left the gel preparation will be dissolved in the receptor fluid. Samples were taken of as much as 2 mL at 30, 90, 150, 210, 270, 330, 390, 450, 510, 570 min and 1, 2, 3, 4, 5, 6, 7, 8, 9, 10 h that determined the drug concentration in the receptor compartment. The sample taken from the receptor compartment is put into a vial as much as 2 mL, and each time the sample is taken it must be replaced with a new receptor fluid with the same volume to keep the volume of fluid in the receptor compartment constant, which is 20 mL. The drug concentration in the donor compartment will decrease and the drug concentration in the receptor compartment will rise to a steady state at a certain time and at a certain concentration. The absorption results obtained were entered into the regression equation and the concentration of the drug penetrated was obtained. Based on the concentration data, the cumulative amount and total flux can be calculated each time the drug is penetrated.

**Figure 9:**
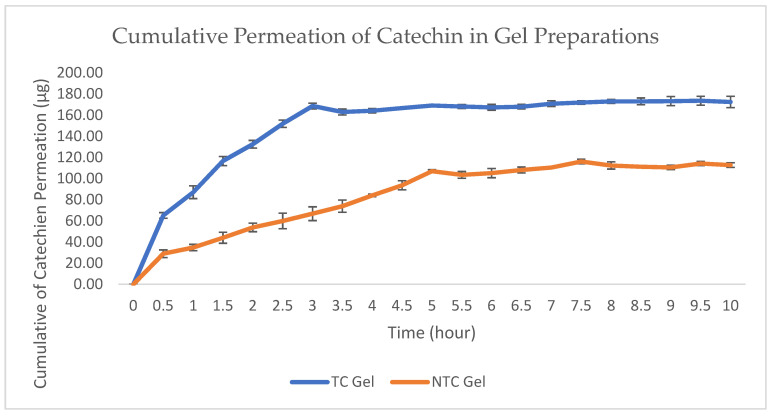
Cumulative permeation of transethosomal catechin gel and non-transethosomal catechin gel.

The cumulative results of catechin penetration through PTFE membranes for 10 h from non-transethosomal gel and transethosomal gel formulations and formulas were 112.741 ± 2.241 μg and 172.454 ± 5.287 μg, respectively. From Figure 9, the cumulative graph of the transethosomal gel and non-transethosomal gel can be seen. The onset or peak time of the two gels was different; the transethosomal gel had a peak at 3 h, while the non-transethosomal gel had a peak at 5.5 h.

Furthermore, the cumulative amount of drug penetrated is plotted at each sampling time. The regression equation obtained from this cumulative amount shows the total flux of the penetrated drug.

**Figure 10:**
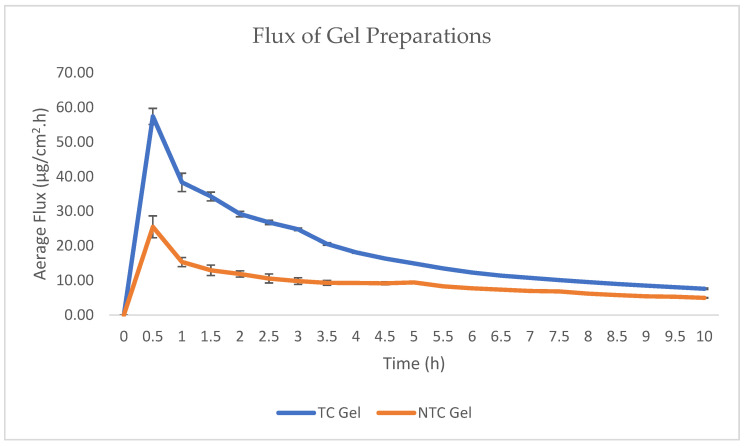
Flux comparison of transethosomal catechin gel and non-transethosomal catechin gel.

The graph in Figure 10 shows the rate or flow rate of catechins that are penetrated in each unit of time. It can be seen that the catechins that penetrated per unit time in both formulas showed a similar profile pattern from 0 to 10 h. However, there is a difference in the number of catechins penetrated.

The flux of drug through the membrane can be affected by its diffusion coefficient across the stratum corneum by interfering with the barrier system of the stratum corneum. To increase the drug flux across the skin membrane, penetration-enhancing compounds can be used [45,46].

According to the cumulative amount and drug flux data, gels with transethosomal carriers had higher flux values and permeability than gel preparations without transethosomes. This is because transethosomes have small particle sizes, contain phospholipids, ethanol, propylene glycol and surfactants that can help penetrate catechins from gel preparations into the skin.

The presence of ethanol as a penetration enhancer will help penetrate the transetosomal gel by increasing the fluidity of lipids in the stratum corneum and reducing the density of lipids in the skin. Therefore, the very soft transethosomal vesicles of the stratum corneum penetrate the structure of the stratum corneum and create a kind of drug absorption pathway in the skin. Phospholipids, as transethosomes, are also one of the factors that help penetrate catechins in the skin by merging with phospholipids in the bilayer membrane of the skin because they have a chemical structure similar to that based on the principle of like dissolves like. In addition, the addition of surfactant tween 80 can form vesicles that are ultra-deformable or more flexible through pores measuring 20–30 nm.

It can be concluded that the factors that can affect drug absorption through the skin are the viscosity of the preparation, the dissolution of a drug in the carrier, the diffusion of the dissolved drug from the carrier to the skin surface, and the penetration of the drug through the skin layer, especially the stratum corneum.

Determination of release kinetics aims to determine the mechanism of drug release from transdermal preparations. Determination of the release kinetics of the preparation of non-transethosomal gel preparations and transethosomal gels was carried out based on the value of the correlation coefficient (r^2^) through a linear equation according to the order. For zero order, the drug release kinetics is determined by plotting the time in hours against the permeation percentage, while for the first order drug release, kinetics is determined by plotting the time value in hours against the logarithm of the permeation percentage. Drug release kinetics of Higuchi model is determined by the root of the square of time to the cumulative percentage of permeation; then, for the Korsmeyer–Peppas model, drug release kinetics is determined by the logarithm of time to the logarithm of the cumulative percentage of permeation.

Based on the correlation coefficient values obtained from plotting the data on the graph, the dominant reaction order in the drug release kinetics can be determined. The most dominant reaction order is indicated by the correlation coefficient value closest to 1 (one). The correlation coefficient data from each preparation are as follows.

At Table 5, both of TCG and NTCG showed that catechin release had Higuchi’s order The Higuchi’s order indicates that in the permeation process of catechins’ passive diffusion occurs and this is in accordance with the theory of transdermal delivery.

### 2.8. Result of Antihypercholesterol in Rats

**Figure 11:**
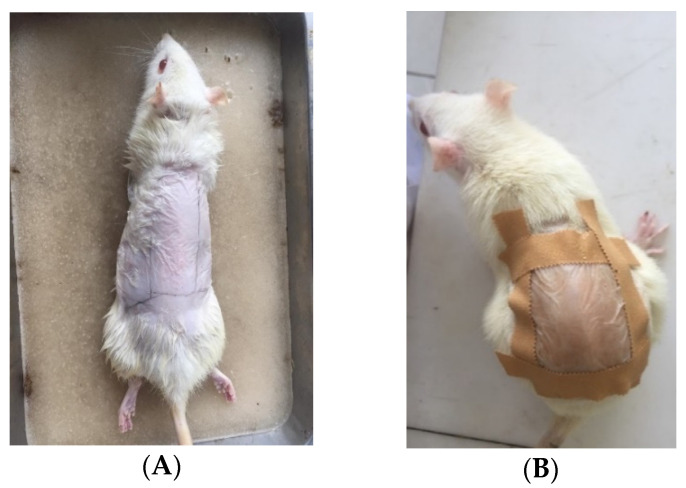
Visualization of experimental animal treatment (**A**) Before gel application, (**B**). During gel application.

TCG and NTCG preparations were applied to the shaved backs of rats, then covered with cellophane plaster (Figure 11). Rats are test animals that are often used and are also suggested in research on the activity of active compounds [47]. The results showed that induction of triton X-100 could increase cholesterol levels in groups of rats, except in the normal group. Hypercholesterol conditions absolutely must occur so that the effect of the treatment given to rats which aims to observe cholesterol reduction can be observed properly. It can be seen that there is a decrease in cholesterol in the parameters of total cholesterol and LDL in the group given oral simvastatin, TCG, and NTCG.

The measurement of total cholesterol was based on an enzymatic reaction using KIT CHOL (LabKit) due to the oxidation of free cholesterol by cholesterol oxidase to form peroxide compounds that affect the oxidative multiplication of phenol and 4-aminoantipyrine (4-AAP) to form a red quinoneimine dye. The chromophore group on the quinoneimine compound provides absorption when read on spectrophotometry, where the light intensity is proportional to the cholesterol concentration of the sample. The measurement results of total cholesterol levels can be seen in Table 6.

Flavonoid compounds have antihypercholesterolemic activity, with their mechanism of action being the reduction of LDL in the body. Flavonoids can also increase the density of LDL receptors in the liver and bind to apolipoprotein B. Flavonoids can reduce cholesterol levels in the blood by inhibiting the work of the HMG Co-A reductase enzyme. The function of HMG Co-A reductase is an enzyme to convert HMG Co-A into mevalonate. Thus, if HMG Co-A reductase is inhibited then mevalonate is inhibited or cannot be formed. The mechanism of these flavonoids is similar to the mechanism of antihyperlipidemic drugs of the statin group or the HMG Co-A reductase inhibitor class [48]. The flavonoid content that plays a major role in reducing triglyceride levels is catechin compounds [49].

Catechins can regulate genes that can metabolize total cholesterol, so that a decrease in LDL levels in blood plasma a catechin effect on lipogenesis gene expression.

**Figure 12:**
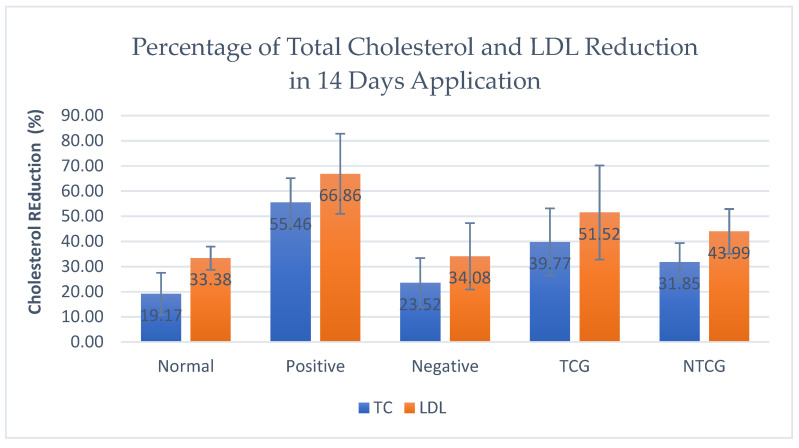
Graph of cholesterol reduction percentage in 14 days application of Total Cholesterol and LD.

The percentage of cholesterol reduction on the two parameters in two weeks is shown in the Figure 12. The positive group showed the largest cholesterol reduction percentage. The TGC group had a higher percentage reduction in total cholesterol and LDL than the NTCG group. Based on these results, it can be concluded that TCG preparations have the potential to be used as antihypercholesterolemia, especially in reducing total cholesterol and LDL. The addition of catechin transethosomes loaded into the gel is necessary to obtain better cholesterol and LDL reduction values. According to statistical calculations of significance between groups obtained, if *p* < 0.05 then there was a significant difference. Therefore, it can be interpreted that in the total cholesterol results there was a significant difference between the normal group and the NTCG group and the normal group with the TCG group (Table 7).

Transethosomes are a better type of UDV compared to the other two types because they contain the advantages possessed by transfersomes and ethosomes. Transethosomes have high ethanol content (30–40%) together with edge activator. The mechanism of skin penetration on transethosomes is a combination of transfersome and ethosomal mechanisms, and is rated higher in studies of vesicle elasticity and permeation through the skin so as to increase the amount of active substance that enters systemically [50]. Ethanol is used as an efficient penetration enhancer in transethosomes because it has an interdigitating effect on the lipid bilayer and increases the fluidity of the stratum corneum lipids [51,52]. Edge activator can damage the transethosomal lipid bilayer and increase the flexibility of the preparation [51,53]. Edge activator can be a surfactant that can lower the interfacial tension and prevent particle aggregation that can occur during storage. Transetosomes can increase drug permeability in the skin to systemic, which can avoid first pass metabolism, controlled drug release, reduce drug side effects, increase pharmacological effects, avoid fluctuations in drug levels, and are comfortable to use so as to improve patient compliance [40].

### 2.9. Histology of Foam Cell

The lipid handling process occurs in macrophages. Endothelial cells have high surface expression of oxidized LDL-1 receptor (LDL) such as a lectin, capable of binding and transferring oxidized LDL (oxLDL) across cells to the intima media, which is infiltrated by macrophages in atherosclerosis. Scavenger receptors such as SR-A1, CD36, and LOX-1 on macrophages can detect and bind to oxLDL. In late endosomes/lysosomes, lysosomal acid lipase (LAL) degrades cholesteryl esters, which are abundant in LDL particles, into free cholesterol and free fatty acids. In the endoplasmic reticulum (ER), cholesterol esters are formed from free cholesterol with the contribution of acyl coenzyme A: cholesterol acyltransferase-1 (ACAT1). Cholesterol esters accumulate in the ER. Neutral cholesteryl ester hydrolase (NCEH) processes cholesterol esters, resulting in free cholesterol that is transported outside the cell via ATP-binding ABC cassette transporters ABCA1 and ABCG1, as well as via SR-BI. Apolipoprotein A-1 (ApoA-1) functions as an acceptor of cholesterol carried by ABCA1. High-density lipoprotein (HDL) accepts cholesterol transferred by ABCG1 and SR-BI. Under normal conditions, this machine is strictly regulated to ensure cholesterol homeostasis. In atherosclerosis, control is deregulated. The expression of scavenger receptors is increased, which leads to increased oxLDL uptake. In contrast, the expression of the cholesterol transporters ABCA1 and ABCG1 is suppressed, which reduces cholesterol efflux and increases cholesterol deposition in macrophages. ACAT1 is upregulated while NCEH is downregulated. This causes the accumulation of cholesterol esters in the cells. Together, these mechanisms lead to excessive lipid deposition and the transformation of macrophages into foam cells [54,55,56].

**Figure 13:**
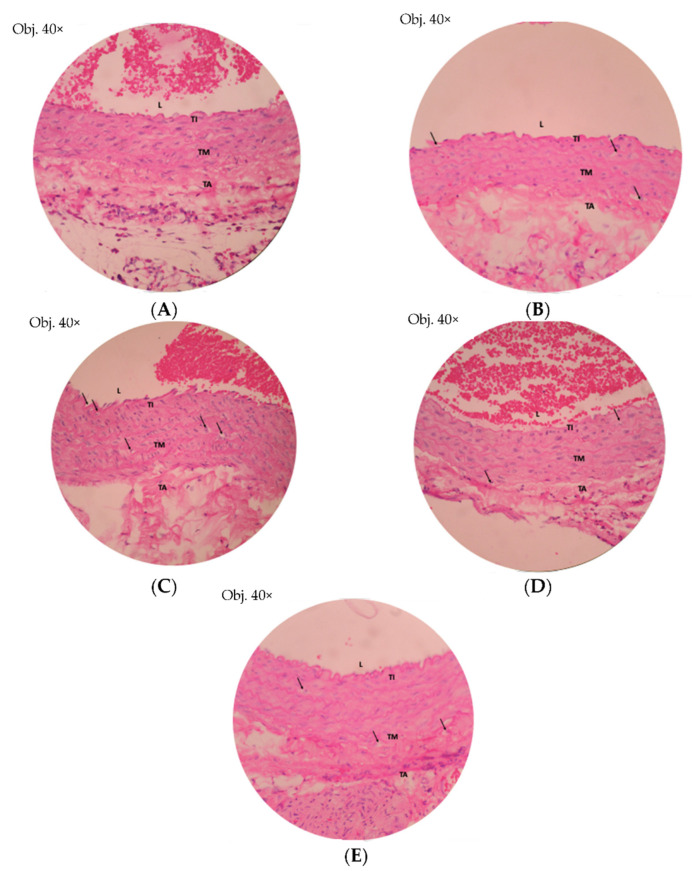
Foam cell in test groups after 2 weeks of treatment. (**A**) Normal, (**B**) Positive, (**C**) Negative, (**D**) TCG, (**E**) NTCG.

In Figure 13, the number of foam cells in rats treated with TCG showed a lower number than foam cells in the negative group. The number of foam cells in the positive group was also less than that in the negative group. The number of foam cells in the NTCG group was found to be higher than in the TCG group. This shows that the administration of oral simvastatin and TCG can reduce the number of foam cells.

## 3. Material and Methods

The purified catechins were obtained at the Central Laboratory of Universitas Padjadjaran and Phospholipon G 90 was purchased from Lipoid International Phar-maceutical Industries Company, Ludwigshafen, Germany. Tween 80, Aethanol and methanol were purchased from Merck, Darmstadt, Germany. Aquades, acetic acid, Carbopol 940, methanol pro analysis, methyl paraben, propylparaben, propylene gly-col, triethanolamine (Merck, Darmstadt, Germany).

### 3.1. Catechin Extract

#### 3.1.1. Extract Purification

The extract purification process was carried out following the stages in the research of Yunarto and Aini, 2005 with modifications [49]. Gambir was grinded and suspended in n-hexane and homogenized using a sonicator for 10 min. The suspension is filtered using filter paper. Then, the residue was dissolved in ethyl acetate and homogenized using a sonicator for 10 min. The ethyl acetate filtrate was partitioned by adding 70 °C (1:1) aquadest, then shaken in a separating funnel and allowed to stand for 30–60 min until two layers formed. The ethyl acetate layer was evaporated using a rotary evaporator until a thick extract was obtained. The purification of gambir extract using HPLC showed that the catechin had a *R*_f_ of 2.534, which was similar to the catechin standard of 2.539. Catechin was identified based on spectroscopic evidence for mass spectroscopic (Figures S1–S3 Supplementary Materials). spectroscopic evidence including infrared (Figures S3–S5 in Supplementary Materials), and ^13^C-NMR (Figures S6–S8 in Supplementary Materials) as well as by comparison with those spectroscopic data previously reported.

#### 3.1.2. Catechin Quantification

Catechin quantification were carried out based on Yunarto and Aini’s research in 2015, with modifications [49]. Catechin standard solutions are made with varying concentrations of 40 ppm, 50 ppm, 60 ppm, 70 ppm, 80 ppm, 90 ppm and 100 ppm in methanol. The standard 100 ppm solution was measured with a UV-Vis spectrophotometer to determine the maximum wavelength of absorption of the catechins. The extract sample solution was made with a concentration of 80 ppm in a methanol solvent. Standard solutions and purified extracts were analyzed using High Performance Liquid Chromatography/HPLC. The HPLC conditions used were C18 column measuring 4.6 × 150 mm, flow rate of 1 mL/min, injection volume of 10 µL and UV detection at the maximum absorption wavelength of catechins (279 nm) using a UV-Vis detector. The mobile phase used isocratic is methanol: water: acetic acid (85:14:1).

### 3.2. Preparation of Transethosomes

Transethosome catechin were prepared using the cold method. Lipophilic phase was made by dissolving phospholipids into half of the amount of ethanol on a magnetic stirrer at 30 °C. Furthermore, the mixed solution is dripped with tween 80 slowly as an edge activator. The hydrophilic phase was made by mixing the remaining ethanol and the weighted buffer. Catechins are dissolved into the solution mixture until dissolved. The two phases are combined and then sonicated using a sonicator probe Hielscher UP200S for 15 min to reduce the vesicle size and stored at 4 °C for further investigation [46].

### 3.3. Characterization of Transethosomes

#### 3.3.1. Determination of Particle Size Distribution, Polydispersity Index, and Zeta Potential

Particle size, polydispersity index and zeta potential of catechin transethosome were characterized by the method Dynamic Light Scattering using a Particle Size Analyzer at 25 ± 1 °C. Samples were diluted and characterized at a scattering angle of 90°. The analysis time was maintained at 60 s, and the mean zeta potential of the vesicles was determined [57].

#### 3.3.2. Determination of Entrapment Efficiency (EE%)

Entrapment Efficiency in transethosomal vesicles was determined by ultracentrifugation at 15,000 rpm and 4 °C for 1 h using a High Speed Refrigerated Centrifuge). After the sample was centrifuged, the supernatant was carefully withdrawn and measured by the method microplate reader.

The entrapment efficiency is calculated using the following equation:

$$\mathrm{Entrapment}\;\mathrm{efficiency}\;\left(\%\right)=\frac{\mathrm{Ao}-\mathrm{Au}}{\mathrm{Ao}}\times 100\%$$

Ao = Total amount of catechins

Au = Measurable amount of catechins in supernatant

The previous step is to make a standard curve of catechins in methanol solvent.

#### 3.3.3. Optimization of the Formulation Ingredients

Before formulating transethosomes, the formula was optimized for transethosomes without catechins, using a two-level factorial design to obtain optimal variations in concentration and formula using Design Expert^®^ 11 (Trial Version) software. The numerical factors selected were phospholipids and tween 80. The variations are showed at Table 8. Phospholipids are important components in the formulation of liposomes [22,58]. Two-level factorial design had a lower (minimum) and an upper (maximum) limit to determine the optimum number of phospholipids and tween 80 to obtain the optimum characteristics of the transethosomal formulation. The lower limit concentration for phospholipids is 3% *w*/*w* and the upper limit is 5% *w*/*w*. Lower limit for tween 80 is 1.5% *w*/*w* and upper limit is 2.5% *w*/*w*.

#### 3.3.4. Catechin Transethosomal Formulation

After obtaining the optimization results from the base formula for transethosomes, catechins with varying concentrations were added to obtain a formula that could best absorb the active substance, and then characterization of the transethosomes was carried out.

#### 3.3.5. Morphological Characterization of the Transethosome

Observations were carried out using the Inverted Microscope Olympus phase contrast, Type CKX 53. The catechin transethosomes were smeared thinly on the surface of the petri dish glass and then observed with various lens magnifications.

Observations were also done using Hitachi TEM System.

### 3.4. Preparation of Catechin Transethosomal Gel

The catechin transethosomal gel was formed by inserting a number of transethosomes containing catechins into an optimized gel base. It is then mixed using a homogenizer at 500 RPM for 15 min [59].

#### 3.4.1. In Vitro Skin Permeation of Catechin from Transethosomal Gel and Non-Transethosomal Gel

The permeation assay uses a modified vertical Franz diffusion cell. The receptor compartment was filled with a mixture of phosphate buffer pH 5.8, then the temperature was maintained at 32 + 0.5 °C and stirred with a stirrer at 100 rpm. Polytetrafluoroethylene (PTFE) membrane was used, and it installed between the donor and receptor compartments. Approximately 2 mL of the sample was withdrawn through the diffusion cell sampling port at intervals of 20 time points. Thereafter, 2 mL of the buffer solution in the receptor compartment was immediately refilled with fresh buffer to maintain sink conditions [44].

#### 3.4.2. Determination of Discharge Kinetics

To determine the mechanism and kinetics of catechin release from the gel in the body, it was carried out by plotting the dissolution results with zero-order kinetics equations, first-order kinetics, Higuchi, and Korsmeyer–Peppas [60].

#### 3.4.3. In Vitro Anticholesterol Activity of Transethosome Catechin Extract

Measurement of antihypercholesterol activity has been carried out using the Liebermann–Burchard method. Each sample was made with a concentration series of 50, 75, 100, 150, and 200ppm from a concentration of 1000 ppm in chloroform p.a solvent. Then, 5 mL of each solution was taken and put into a test tube; then, we added 5 mL of standard cholesterol solution with a concentration of 400 ppm in chloroform p.a and shook for 2 min. Then, 5 mL of the mixture was taken, and 2 mL of anhydrous acetic acid and 0.1 mL of concentrated H_2_SO_4_ were added. The solution was allowed to stand in a dark place for 30 min, until a green color change was formed. The color results obtained were read with the UV-Vis spectrophotometer at its maximum wavelength.

The blank solution was 5 mL chloroform p.a plus 2 mL anhydrous acetic acid and 0.1 mL concentrated H_2_SO_4_. Meanwhile, the negative control used was 5 mL of 400 ppm cholesterol solution in acetone p.a plus 2 mL of anhydrous acetic acid and 0.1 mL of concentrated H_2_SO_4_.

#### 3.4.4. In Vivo Anticholesterol Activity of Trasethosome Catechin Extract

The activity was tested for 28 days. In the first 14 days, the rats were given triton X-100 to reach the hyperlipidemia condition, and the next 14 days were given the test treatment according to the group’s treatment of the test animals. Lipid profile measurements were carried out on days 0, 14, 21 and 28. Samples were centrifuged at 9000 RPM for 15 min to separate serum from blood. After that, lipid profiles such as Total Cholesterol (TC) and LDL in mg/dL. Measurement of lipid profile using a LAB KIT reagents and analysis by UV-VIS spectrophotometry at a wavelength of 505 nm [61].

#### 3.4.5. Animal Treatment

An application letter for the ethical use of experimental animals was approved from the Research Ethics Commission (KEP) Universitas Padjadjaran in Bandung on 5 April 2021 and obtained ethical approval letter no. 262/UN6.KEP/EC/2021.

Rats are animals that are widely used and are recommended in in vivo tests [47]. Rats were acclimatized for 7 days. Rats were given ketamine anesthesia at a dose of 100 mg/kg. Next, 1.5 mL of blood was drawn from the mice using a capillary tube through the retro orbital sinus. On the experimental day, triton was administered at a dose of 40 mg/kg in saline solution once a day intraperitoneally. The group was divided into five (Table 9). It based on the treatment received by the rats as the animal test.

One day before being given treatment, the rats were shaved on the back with an area of 3 × 3 cm using an animal hair clipper (Kemei^®^ Professional Pet Clipper). On the 7th and 14th days, the rats were again given ketamine anesthesia with a dose of 100 mg/kg. Furthermore, 1.5 mL of blood was drawn from mice using a capillary tube through the retro orbital sinus to measure the lipid profile. Then, the experiment was continued for 14 days for administration of the preparation. Normal controls and negative controls were only orally given carboxy methyl cellulose (CMC). For the positive control, the rats were given the drug simvastatin orally at a dose of 10 mg/kg with the help of an oral probe. For the control of the test, the rats were given transethosomal and non-transethosomal gel preparations on the backs of the rats that had been marked with a box, covered with a transparent plaster and left for 4 h. After that, they were rinsed with water [46].

## 4. Conclusions

The optimum blank formula for transethosomal preparations based on a two-level factorial design method was formula with a composition of 3% phospholipid and 2.5% tween 80. This formula has the highest desirability value of 0.664 so it can be chosen to be loaded with purified catechin. The preparation of transethosomal catechins has good characteristics and complies with the requirements on particle size, polydispersity index and entrapment efficiency. However, the stability of this preparation does not meet the requirements for acceptance of the zeta potential. The optimum catechin transethosomal formula based on the characterization results was formula with 1% of catechin. This formula obtains the best particle size and entrapment efficiency compared to other formulas. The results of the in vitro permeation test showed the similarity of the permeation profiles of the non-transtosome gel and the transtosome gel, but there was a difference between the cumulative volume of drugs penetrated into the system. Cumulative catechins penetrated from TC gels were greater than NTC gels.

The results of the antihypercholesterol activity test for 14 days in rats showed that there was a better decrease in cholesterol values in the application of TC gel compared to NTC gel.

## Figures and Tables

**Table 1:** Upper and Lower Limit Values in Phospholipids and Tween 80.

Factor	Lower Limit (%)	Upper Limit (%)
**Phospholipids**	3	5
**Tween 80**	1.5	2.5

**Table 2:** The result of optimization blank transethosom.

Formulation	Particle Size (nm)	Polydispersity Index
Fa	225.23 ± 22.9	0.326 ± 0.2
Fb	139.3 ± 8.5	0.225 ± 0.07
Fc	676.16 ± 52.9	0.538 ± 0.02
Fd	390.5 ± 4.0	0.515 ± 0.02

**Fa** = Phospolipid 3%; Tween 80 1.5%; **Fb** = Phospolipid 3%; Tween 80 2.5%; **Fc**= Phospolipid 5%; Tween 80 1.5%; **Fd** = Phospolipid 5%; Tween 80 2.5%.

**Table 3:** Desirability Value Based on Expert Design.

No.	Phospholipid	Tween 80	Particle Size	Polydispersity Index	Desirability
1	3	2.5	139.3	0.226	0.664
2	3.022	2.5	142.014	0.229	0.655
3	3	2.452	143.409	0.230	0.650
4	3	2.234	162.127	0.252	0.584

**Table 4:** Characterization results of catechin transethosomes.

Parameters	A	B	C	D
Particle Size (nm)	155.9 ± 5.7	161.83 ± 1.5	176.1 ± 5.8	219.53 ± 9.23
Polydispersity Index	0.134 ± 0.02	0.151 ± 0.09	0.11 ± 0.11	0.276 ± 0.05
Zeta Potential (mV)	−9.36 ± 5.08	−12.63 ± 3.52	−11.6 ± 5.28	−8.86 ± 2.93
Entrapment Efficiency (%)	93.58 ± 0.01	95.71 ± 0.005	96.77 ± 0.005	97.43

Note: each value is expressed as mean ± standard deviation (*n* = 3). **A** = catechin 0.5%; **B** = catechin 0.75%; **C** = catechin 1%; **D** = catechin 1.25%.

**Table 5:** Coefficient correlation of TCG and NTCG.

Preparation	Correlation Coefficient Value (r^2^)			
	Zero Order	First Order	Higuchi	Korsmeyer–Peppas
Figure 11	0.8464	0.0909	**0.9532**	0.6599
TCG	0.5504	0.0886	**0.7844**	0.3793

**Table 6:** Result of TC (**a**) and LDL (**b**) during treatment.

**TC**	**T1 (mg/dL)**	**T2 (mg/dL)**	**T1-T2 (mg/dL)**	**(T1-T2)/T1 (mg/dL)**	**% (b)**	**Average (c)**	**SD (c)**
			**(a)**	**(b)**	**(c)**	**(d)**	**(e)**
Normal	171.69	133.41	38.28	0.22	22.30	19.17	8.40
	196.75	159.63	37.12	0.19	18.87		
	153.13	144.78	8.35	0.05	5.45		
	169.37	133.64	35.73	0.21	21.10		
	244.08	175.41	68.68	0.28	28.14		
Positive	270.33	120.56	149.77	0.55	55.40	55.46	9.63
	275.41	124.59	150.82	0.55	54.76		
	224.83	79.81	145.02	0.65	64.50		
	242.00	90.49	151.51	0.63	62.61		
	322.74	193.50	129.24	0.40	40.04		
Negative	332.48	258.47	74.01	0.22	22.26	23.52	9.86
	276.80	238.28	38.52	0.14	13.92		
	184.87	146.72	38.15	0.21	20.64		
	284.92	171.00	113.92	0.40	39.98		
	196.98	158.47	38.51	0.20	19.55		
TCG	261.95	189.56	72.39	0.28	27.64	39.77	13.36
	322.97	167.98	154.99	0.48	47.99		
	286.31	131.79	154.52	0.54	53.97		
	195.82	149.65	46.17	0.24	23.58		
	266.13	144.55	121.58	0.46	45.68		
NTCG	273.32	201.86	71.46	0.26	26.15	31.85	7.52
	245.24	159.34	85.90	0.35	35.03		
	245.71	165.66	80.05	0.33	32.58		
	231.55	177.73	53.82	0.23	23.24		
	256.84	148.26	108.58	0.42	42.28		
**LDL**	**T1 (mg/dL)**	**T2 (mg/dL)**	**T1-T2 (mg/dL)**	**(T1-T2)/T1 (mg/dL)**	**%(b)**	**Average (c)**	**SD (c)**
			**(a)**	**(b)**	**(c)**	**(d)**	**(e)**
Normal	63.06	39.44	23.63	0.37	37.46	33.38	4.63
	72.41	47.90	24.51	0.34	33.84		
	90.78	67.27	23.52	0.26	25.90		
	110.69	69.84	40.85	0.37	36.90		
	125.54	84.39	41.15	0.33	32.78		
Positive	178.67	58.77	11.90	0.67	67.11	66.86	15.95
	121.12	64.03	57.10	0.47	47.14		
	161.66	13.52	148.14	0.92	91.64		
	182.53	65.67	116.86	0.64	64.02		
	207.67	73.97	133.70	0.64	64.38		
Negative	262.91	182.85	80.05	0.30	30.45	34.08	13.16
	141.96	83.11	58.85	0.41	41.46		
	121.56	78.92	42.64	0.35	35.08		
	197.94	100.53	97.41	0.49	49.21		
	107.31	92.06	15.24	0.14	14.21		
TCG	167.73	80.37	87.36	0.52	52.08	51.52	18.71
	254.09	85.74	168.36	0.66	66.26		
	197.68	71.46	126.22	0.64	63.85		
	95.06	42.10	52.95	0.56	55.71		
	91.13	73.18	17.95	0.20	19.69		
NTCG	159.42	106.35	53.07	0.33	33.29	43.99	8.89
	116.57	68.70	47.87	0.41	41.07		
	147.10	63.05	84.05	0.57	57.14		
	167.66	98.84	68.83	0.41	41.05		
	152.30	80.13	72.17	0.47	47.39		

**Table 7:** The results of statistical significance calculations between test groups.

Group	Significance	
	TC	LDL
Normal-NTCG	0.023 *	0.078
Normal-TCG	0.007 *	0.025 *
Negative-NTCG	0.229	0.699
Negative-TCG	0.103	0.390
NTCG-Positive	0.264	0.144
TCG-Positive	0.492	0.323
NTCG-TCG	0.667	0.636

* *p* < 0.05 considered for significance.

**Table 8:** Formulas for optimization transethosomal catechin.

Ingredients	Concentration *w*/*w* (%)			
	F1	F2	F3	F4
**Phospholipid**	3	3	5	5
**Tween 80**	1.5	2.5	1.5	2.5
**Ethanol**	40	40	40	40
**Acetate Buffer (pH 5.5)**	Ad 100	Ad 100	Ad 100	Ad 100

**Table 9:** Group of animal testing.

Group	Treatment
Normal	Carboxy Methyl Cellulose (CMC) administration orally
Positive	Triton induction 40 mg/kg BW + Simvastatin 10 mg/kg BW orally
Negative	Triton induction 40 mg/kg BW intraperitoneally
TCG	Triton induction 40 mg/kg BW + TCG
NTCG	Triton induction 40 mg/kg BW + NTCG

## Data Availability

Not applicable.

## Supplementary Materials

The following supporting information can be downloaded at: https://www.mdpi.com/article/10.3390/ph15091142/s1, Figure S1: MS Spectrum Prediction; Figure S2: MS Spectrum; Figure S3: IR Spectrum of Catechin Standard; Figure S4: IR Spectrum Purified Catechin; Figure S5: Spectrum IR Transethosomal Catechin; Figure S6: NMR-C Spectrum; Figure S7: NMR Spectrum DEPT; Figure S8: NMR Overlay Spectrum; Figure S9: PSA PDI Zeta Potential of Transethosome Catechin.

## References

[1] Bhatnagar D., Soran H., Durrington P.N. (2008). Hypercholesterolaemia and its management. BMJ.

[2] Karr S. (2017). Epidemiology and management of hyperlipidemia. Am. J. Manag. Care..

[3] Golomb B.A., Evans M.A. (2008). Statin adverse effects: A review of the literature and evidence for a mitochondrial mechanism. Am. J. Cardiovasc. Drugs.

[4] Walters K.A. (2002). Dermatological and Transdermal Formulations.

[5] Wu N., Sarna L.K., Hwang S.Y., Zhu Q., Wang P., Siow Y.L., Karmin O. (2013). Activation of 3-hydroxy-3-methylglutaryl coenzyme A (HMG-CoA) reductase during high fat diet feeding. Biochim. Biophys. Acta Mol. Basis Dis..

[6] Ngamukote S., Mäkynen K., Thilawech T., Adisakwattana S. (2011). Cholesterol-Lowering Activity of the Major Polyphenols in Grape Seed. Molecules.

[7] Adelina R. (2018). Mekanisme Katekin Sebagai Obat Antidislipidemia (Uji In Silico). Bul. Penelit. Kesehat..

[8] Isemura M. (2019). Catechin in Human Health and Disease. Molecules.

[9] Kemenkes R.I. (2017). Farmakope Herbal Indonesia Edisi 2. Kementrian Kesehatan. Republik Indnesia.

[10] Ravipati A.S., Reddy N., Koyyalamudi S.R. (2014). Biologically Active Compounds from the Genus Uncaria (Rubiaceae). Stud. Nat. Prod. Chem..

[11] Yunarto N., Sulistyaningrum N., Kurniatri A.A., Elya B. (2021). Gambir (*Uncaria gambir* Roxb.) as A Potential Alternative Treatment for Hyperlipidemia. Media Penelit. Dan Pengemb. Kesehat..

[12] Saad M.F.M., Goh H.H., Rajikan R., Yusof T.R.T., Baharum S.N., Bunawan H. (2020). Uncaria gambir (W. Hunter) Roxb.: From phytochemical composition to pharmacological importance. Trop. J. Pharm. Res..

[13] Rauf A., Rahmawaty, Siregar A.Z. (2015). The Condition of Uncaria gambir Roxb. as One of Important Medicinal Plants in North Sumatra Indonesia. Procedia Chem..

[14] Aprely K.J., Misfadhila S., Asra R. (2021). A Review: The Phytochemistry, Pharmacology and Traditional Use of Gambir (*Uncaria gambir* (Hunter) Roxb.). EAS J. Pharm. Pharmacol..

[15] Gadkari P.V., Balaraman M. (2015). Catechins: Sources, extraction and encapsulation: A review. Food Bioprod. Process..

[16] Park Y., Park E.-M., Kim E.-H., Chung I.-M. (2014). Hypocholesterolemic metabolism of dietary red pericarp glutinous rice rich in phenolic compounds in mice fed a high cholesterol diet. Nutr. Res. Pract..

[17] Sergent T., Vanderstraeten J., Winand J., Beguin P., Schneider Y.-J. (2012). Phenolic compounds and plant extracts as potential natural anti-obesity substances. Food Chem..

[18] Cai Z.-Y., Li X.-M., Liang J.-P., Xiang L.-P., Wang K.-R., Shi Y.-L., Yang R., Shi M., Ye J.-H., Lu J.-L. (2018). Bioavailability of Tea Catechins and Its Improvement. Molecules.

[19] Kil Song C., Balakrishnan P., Shim C.-K., Chung S.-J., Chong S., Kim D.-D. (2012). A novel vesicular carrier, transethosome, for enhanced skin delivery of voriconazole: Characterization and in vitro/in vivo evaluation. Colloids Surf. B Biointerfaces.

[20] Megha J., Ganesh N.S., Chandy V. (2021). Transethosome: A Versatile Carrier for Enhanced Transdermal Delivery. Int. Res. J. Pharm. Biosci..

[21] Azzahra L., Mita S.R., Sriwidodo S. (2020). Formulation, Characterization, and Herbal Drug Delivery Applications of Ethosome, Transfersome, and Transethosome Luthfia. Indones. J. Pharm..

[22] Natsheh N., Touitou E. (2020). Phospholipid Vesicles for Dermal/Transdermal and Nasal Administration of Active Molecules: The Effect of Surfactants and Alcohols on the Fluidity of Their Lipid Bilayers and Penetration. Molecules.

[23] Homayun B., Lin X., Choi H.-J. (2019). Challenges and Recent Progress in Oral Drug Delivery Systems for Biopharmaceuticals. Pharmaceutics.

[24] Lu M., Qiu Q., Luo X., Liu X., Sun J., Wang C., Lin X., Deng Y., Song Y. (2019). Phyto-phospholipid complexes (phytosomes): A novel strategy to improve the bioavailability of active constituents. Asian J. Pharm. Sci..

[25] Liu X., Zhang R., McClements D.J., Li F., Liu H., Cao Y., Xiao H. (2018). Nanoemulsion-Based Delivery Systems for Nutraceuticals: Influence of Long-Chain Triglyceride (LCT) Type on In Vitro Digestion and Astaxanthin Bioaccessibility. Food Biophys..

[26] Quagliariello V., Vecchione R., Coppola C., Di Cicco C., De Capua A., Piscopo G., Paciello R., Narciso V., Formisano C., Taglialatela-Scafati O. (2018). Cardioprotective Effects of Nanoemulsions Loaded with Anti-Inflammatory Nutraceuticals against Doxorubicin-Induced Cardiotoxicity. Nutrients.

[27] Baba M.A.W.N. (2014). Nutraceutical Properties of the Green Tea Polyphenols. J. Food Process. Technol..

[28] Manconi M., Marongiu F., Castangia I., Manca M.L., Caddeo C., Tuberoso C.I.G., D’Hallewin G., Bacchetta G., Fadda A.M. (2016). Polymer-associated liposomes for the oral delivery of grape pomace extract. Colloids Surf. B Biointerfaces.

[29] Shinji S., Yasukazu T., Hatsue W., Kazuo K., Machiko I., Naoki M. (2011). Analysis of brain cell activation by nanosized particles of Ginkgo biloba extract. Int. J. Plant Physiol. Biochem..

[30] Abdelwahab S.I., Taha M.M.E., Sheikh B.Y., How C.W., El-Sunousi R., Abdullah R., Eid E.E., Yagoub U. (2013). Thymoquinone-loaded nanostructured lipid carriers: Preparation, gastroprotection, in vitro toxicity, and pharmacokinetic properties after extravascular administration. Int. J. Nanomed..

[31] Yeung A.W.K., Souto E.B., Durazzo A., Lucarini M., Novellino E., Tewari D., Wang D., Atanasov A.G., Santini A. (2020). Big impact of nanoparticles: Analysis of the most cited nanopharmaceuticals and nanonutraceuticals research. Curr. Res. Biotechnol..

[32] Pimentel-Moral S., Teixeira M.C., Fernandes A.R., Borrás-Linares I., Arráez-Román D. (2019). Polyphenols-enriched Hibiscus sabdariffa extract-loaded nanostructured lipid carriers (NLC): Optimization by multi-response surface methodology. J. Drug Deliv. Sci. Technol..

[33] Peram M.R., Jalalpure S.S., Kumbar V.M., Patil S.R., A Joshi S., Bhat K.G., Diwan P.V. (2019). Factorial design based curcumin ethosomal nanocarriers for the skin cancer delivery: In vitro evaluation. J. Liposome Res..

[34] Komatsu Y., Suematsu S., Hisanobu Y., Saigo H., Matsuda R., Hara K. (1993). Effects of pH and Temperature on Reaction Kinetics of Catechins in Green Tea Infusion Effects of pH and Temperature on Reaction Kinetics of Catechins in Green Tea Infusion. Biosci. Biotechnol. Biochem..

[35] Li N., Taylor L.S., Ferruzzi M.G., Mauer L.J. (2012). Kinetic Study of Catechin Stability: Effects of pH, Concentration, and Temperature. J. Agric. Food Chem..

[36] Yeni G., Syamsu K., Suparno O., Mardliyati E., Muchtar H. (2014). Repeated extraction process of raw gambiers (*Uncaria gambier* Robx.) for the catechin production as an antioxidant. Int. J. Appl. Eng. Res..

[37] Yu Y.-Q., Yang X., Wu X.-F., Fan Y.-B. (2021). Enhancing Permeation of Drug Molecules Across the Skin via Delivery in Nanocarriers: Novel Strategies for Effective Transdermal Applications. Front. Bioeng. Biotechnol..

[38] Luo Y., Wang T.T.Y., Teng Z., Chen P., Sun J., Wang Q. (2013). Encapsulation of indole-3-carbinol and 3,3′-diindolylmethane in zein/carboxymethyl chitosan nanoparticles with controlled release property and improved stability. Food Chem..

[39] Weng J., Tong H.H.Y. (2020). In Vitro Release Study of the Polymeric Drug Nanoparticles: Development and Validation of a Novel Method. Pharmaceutics.

[40] Ascenso A., Raposo S., Batista C., Cardoso P., Mendes T., Praça F.G., Bentley M.V., Simões S. (2015). Development, characterization, and skin delivery studies of related ultradeformable vesicles: Transfersomes, ethosomes, and transethosomes. Int. J. Nanomed..

[41] Honary S., Zahir F. (2013). Effect of zeta potential on the properties of nano-drug delivery systems—A review (Part 1). Trop. J. Pharm. Res..

[42] Mota A., Prazeres I., Mestre H., Bento-Silva A., Rodrigues M., Duarte N., Serra A., Bronze M., Rijo P., Gaspar M. (2021). A Newfangled Collagenase Inhibitor Topical Formulation Based on Ethosomes with *Sambucus nigra* L. Extract. Pharmaceuticals.

[43] Lukić M., Pantelić I., Savić S. (2021). Towards Optimal pH of the Skin and Topical Formulations: From the Current State of the Art to Tailored Products. Cosmetics.

[44] I.R. The United States Pharmacopeial Convention (2014). ⟨1724⟩ Semisolid Drug Products—Performance Tests. Usp.

[45] Briuglia M.-L., Rotella C.M., McFarlane A., Lamprou D.A. (2015). Influence of cholesterol on liposome stability and on in vitro drug release. Drug Deliv. Transl. Res..

[46] Abdulbaqi I.M., Darwis Y., Assi R.A., Khan N.A.K. (2018). Transethosomal gels as carriers for the transdermal delivery of colchicine: Statistical optimization, characterization, and ex vivo evaluation. Drug Des. Dev. Ther..

[47] Sharma N., Agarwal G., Rana A.C., Bhat Z.A., Kumar D. (2011). A review: Transdermal drug delivery system: A tool for novel drug delivery system. Int. J. Drug Dev. Res..

[48] Sutjiatmo A.B., Sukandar E.Y., Sinaga R., Hernawati R., Vikasari S.N. (2013). Efek antikolesterol ekstrak etanol daun cermE (*Phyllanthus acidus* (L.) Skeels) pada tikus wistar betina. Kartika J. Ilm. Farm..

[49] Yunarto N., Elya B., Konadi L. (2015). Potensi Fraksi Etil Asetat Ekstrak Daun Gambir (*Uncaria gambir* Roxb.) sebagai Antihiperlipidemia Potency of Ethyl Acetate Fraction of Gambier Leaves Extract Abstrak mengandung katekin adalah gambir Alat dan bahan ini adalah rotary evaporator (Buchi). J. Kefarmasian Indones..

[50] Sudhakar K., Fuloria S., Subramaniyan V., Sathasivam K.V., Azad A.K., Swain S.S., Sekar M., Karupiah S., Porwal O., Sahoo A. (2021). Ultraflexible Liposome Nanocargo as a Dermal and Transdermal Drug Delivery System. Nanomaterials.

[51] Shaji J., Bajaj R. (2018). Transethosomes: A New Prospect for Enhanced Transdermal Delivery. Int. J. Pharm. Sci. Res..

[52] Kumar L., Utreja P. (2019). Formulation and Characterization of Transethosomes for Enhanced Transdermal Delivery of Propranolol Hydrochloride. Micro Nanosyst..

[53] Deepika P., Reathore K.S. (2021). Novel and Most Prominent Carrier System Transethosome for Topical Delivery. Pharm. Reson..

[54] Chistiakov D.A., Melnichenko A.A., Myasoedova V.A., Grechko A.V., Orekhov A.N. (2017). Mechanisms of foam cell formation in atherosclerosis. J. Mol. Med..

[55] Volobueva A., Zhang D., Grechko A.V., Orekhov A.N. (2018). ScienceDirect Review article Foam cell formation and cholesterol trafficking and metabolism disturbances in atherosclerosis. Cor. Vasa.

[56] Yu X., Fu Y., Zhang D., Yin K., Tang C. (2013). Foam cells in atherosclerosis. Clin. Chim. Acta.

[57] Ahad A., Al-Saleh A.A., Al-Mohizea A.M., Al-Jenoobi F.I., Raish M., Yassin A.E.B., Alam M.A. (2017). Formulation and characterization of novel soft nanovesicles for enhanced transdermal delivery of eprosartan mesylate. Saudi Pharm. J..

[58] Singh R.P., Gangadharappa H.V., Mruthunjaya K. (2017). Phospholipids: Unique carriers for drug delivery systems. J. Drug Deliv. Sci. Technol..

[59] Azizah N., Sagita E., Iskandarsyah I. (2017). In vitro penetration tests of transethosome gel preparations containing capsaicin. Int. J. Appl. Pharm..

[60] Wojcik-Pastuszka D., Krzak J., Macikowski B., Berkowski R., Osiński B., Musiał W. (2019). Evaluation of the Release Kinetics of a Pharmacologically Active Substance from Model Intra-Articular Implants Replacing the Cruciate Ligaments of the Knee. Materials.

[61] Raj B., Chandrasekhar K.B., Reddy K.B. (2018). In-vivo Pharmacodynamic studies of Simvastatin NLC loaded Transdermal Patch. Iaetsd J. Adv. Res. Appl. Sci..

