# Supplementary material for: Nanoparticulate System for the Transdermal Delivery of Catechin as an Antihypercholesterol: In Vitro and In Vivo Evaluations
Source: Pharmaceuticals (Basel). 2022 Sep 13;15(9):1142. doi: 10.3390/ph15091142 (PMC9505170; doi:10.3390/ph15091142)
Supplement: Supplementary file 1 [file pharmaceuticals-15-01142-s001.zip › Figure S7. NMR Spectrum DEPT.pdf]

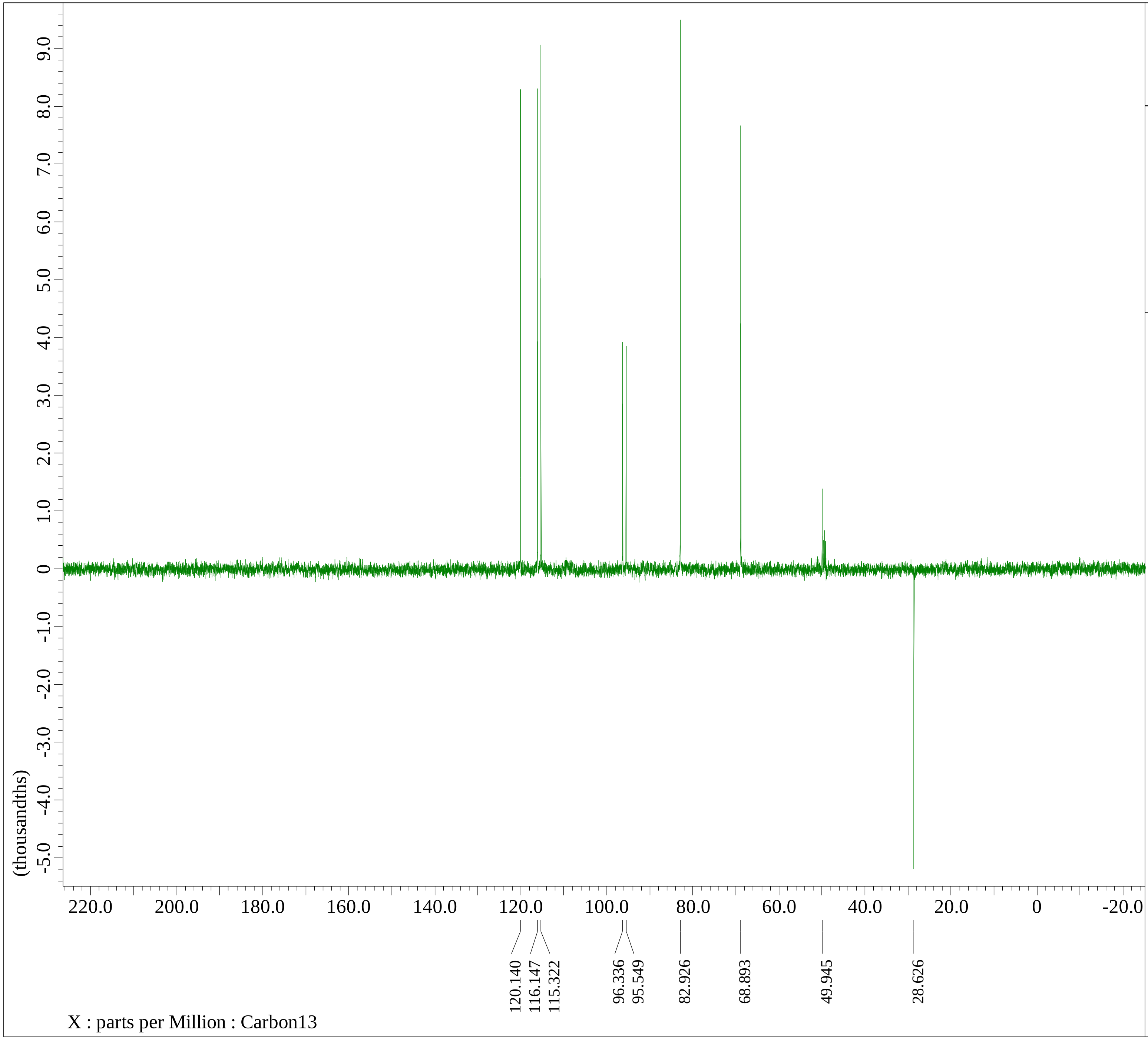


```
---- PROCESSING PARAMETERS ----
sexp( 2.0[Hz], 0.0[s] )
trapezoid( 0[%], 0[%], 80[%], 100[%] )
zerofill( 1 )
fft( 1, TRUE, TRUE )
machinephase
peak_pick( 0[Hz], 50[Hz], Peaks, 0[Hz] )
ppm

Derived from: SORAYA-1_DEPT135deg-1-1.jdf
```

```
Filename                    = SORAYA-1_DEPT135de
Author                      = delta
Experiment                  = dept.jxp
Sample_Id                   = SORAYA-1
Solvent                     = METHANOL-D4
Actual_Start_Time           =  2-JAN-2020 13:03:
Revision_Time               =  2-JAN-2020 15:58:

Comment                     = DEPT with decoupli
Data_Format                 = 1D COMPLEX
Dim_Size                    = 26214
X_Domain                    = Carbon13
Dim_Title                   = Carbon13
Dim_Units                   = [ppm]
Dimensions                  = X
Spectrometer                = JNM-ECZ500R/S1

Field_Strength              = 11.7473579[T] (500
X_Acq_Duration              = 0.82837504[s]
X_Domain                    = Carbon13
X_Freq                      = 125.76529768[MHz]
X_Offset                    = 100[ppm]
X_Points                    = 32768
X_Prescans                  = 4
X_Resolution                = 1.20718268[Hz]
X_Sweep                     = 39.55696203[kHz]
X_Sweep_Clipped             = 31.64556962[kHz]
Irr_Domain                  = Proton
Irr_Freq                    = 500.15991521[MHz]
Irr_Offset                  = 5.0[ppm]
Blanking                    = 2[us]
Clipped                     = TRUE
Scans                       = 2000
Total_Scans                 = 2000

Relaxation_Delay            = 2[s]
Recvr_Gain                  = 56
Temp_Get                    = 17.9[dC]
X_Acq_Time                  = 0.82837504[s]
X_Atn                       = 11[dB]
X_Pulse                     = 11.3[us]
Irr_Atn                     = 8.9[dB]
Irr_Atn_Dec                 = 30.11[dB]
Irr_Atn_Dec_Calc            = 30.11[dB]
Irr_Atn_Dec_Default_Calc    = 30.11[dB]
Irr_Dec_Bandwidth_Hz        = 5.97826087[kHz]
Irr_Dec_Bandwidth_Ppm       = 11.9526989[ppm]
Irr_Dec_Freq                = 500.15991521[MHz]
```

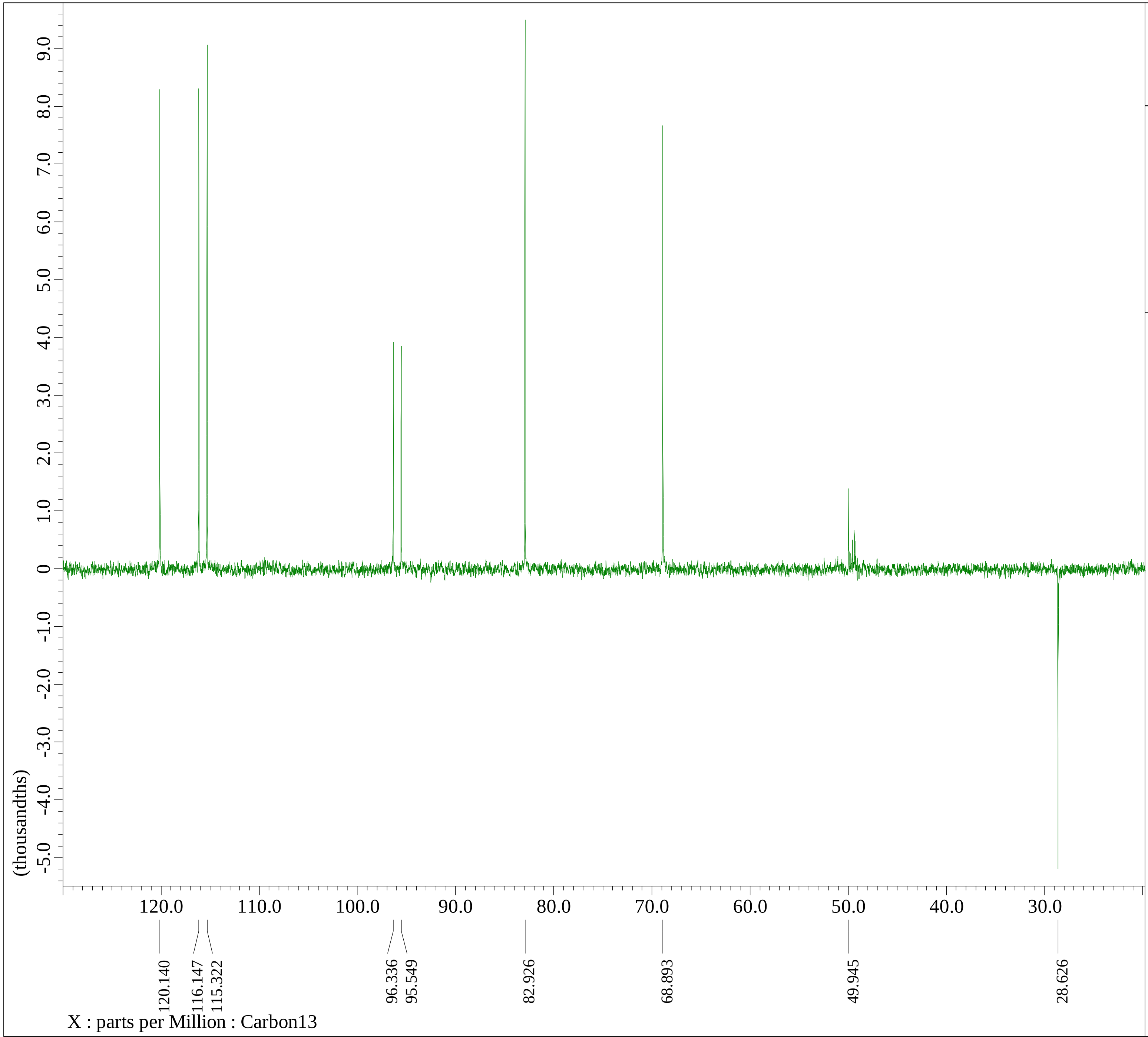

---- PROCESSING PARAMETERS ----
sexp(2.0[Hz], 0.0[s])
trapezoid(0[%], 0[%], 80[%], 100[%])
zerofill(1)
fft(1, TRUE, TRUE)
machinephase
peak_pick(0[Hz], 50[Hz], Peaks, 0[Hz])
ppm

Derived from: SORAYA-1_DEPT135deg-1-1.jdf

Filename	= SORAYA-1_DEPT135de
Author	= delta
Experiment	= dept.jxp
Sample_Id	= SORAYA-1
Solvent	= METHANOL-D4
Actual_Start_Time	= 2-JAN-2020 13:03:
Revision_Time	= 2-JAN-2020 15:58:
Comment	= DEPT with decoupli
Data_Format	= 1D COMPLEX
Dim_Size	= 26214
X_Domain	= Carbon13
Dim_Title	= Carbon13
Dim_Units	= [ppm]
Dimensions	= X
Spectrometer	= JNM-ECZ500R/S1
Field_Strength	= 11.7473579[T] (500
X_Acq_Duration	= 0.82837504[s]
X_Domain	= Carbon13
X_Freq	= 125.76529768[MHz]
X_Offset	= 100[ppm]
X_Points	= 32768
X_Prescans	= 4
X_Resolution	= 1.20718268[Hz]
X_Sweep	= 39.55696203[kHz]
X_Sweep_Clipped	= 31.64556962[kHz]
Irr_Domain	= Proton
Irr_Freq	= 500.15991521[MHz]
Irr_Offset	= 5.0[ppm]
Blanking	= 2[us]
Clipped	= TRUE
Scans	= 2000
Total_Scans	= 2000
Relaxation_Delay	= 2[s]
Recvr_Gain	= 56
Temp_Get	= 17.9[dC]
X_Acq_Time	= 0.82837504[s]
X_Atn	= 11[dB]
X_Pulse	= 11.3[us]
Irr_Atn	= 8.9[dB]
Irr_Atn_Dec	= 30.11[dB]
Irr_Atn_Dec_Calc	= 30.11[dB]
Irr_Atn_Dec_Default_Calc	= 30.11[dB]
Irr_Dec_Bandwidth_Hz	= 5.97826087[kHz]
Irr_Dec_Bandwidth_Ppm	= 11.9526989[ppm]
Irr_Dec_Freq	= 500.15991521[MHz]
