# Supplementary material for: Nanoparticulate System for the Transdermal Delivery of Catechin as an Antihypercholesterol: In Vitro and In Vivo Evaluations
Source: Pharmaceuticals (Basel). 2022 Sep 13;15(9):1142. doi: 10.3390/ph15091142 (PMC9505170; doi:10.3390/ph15091142)
Supplement: Supplementary file 1 [file pharmaceuticals-15-01142-s001.zip › Figure S6. NMR-C Spectrum.pdf]

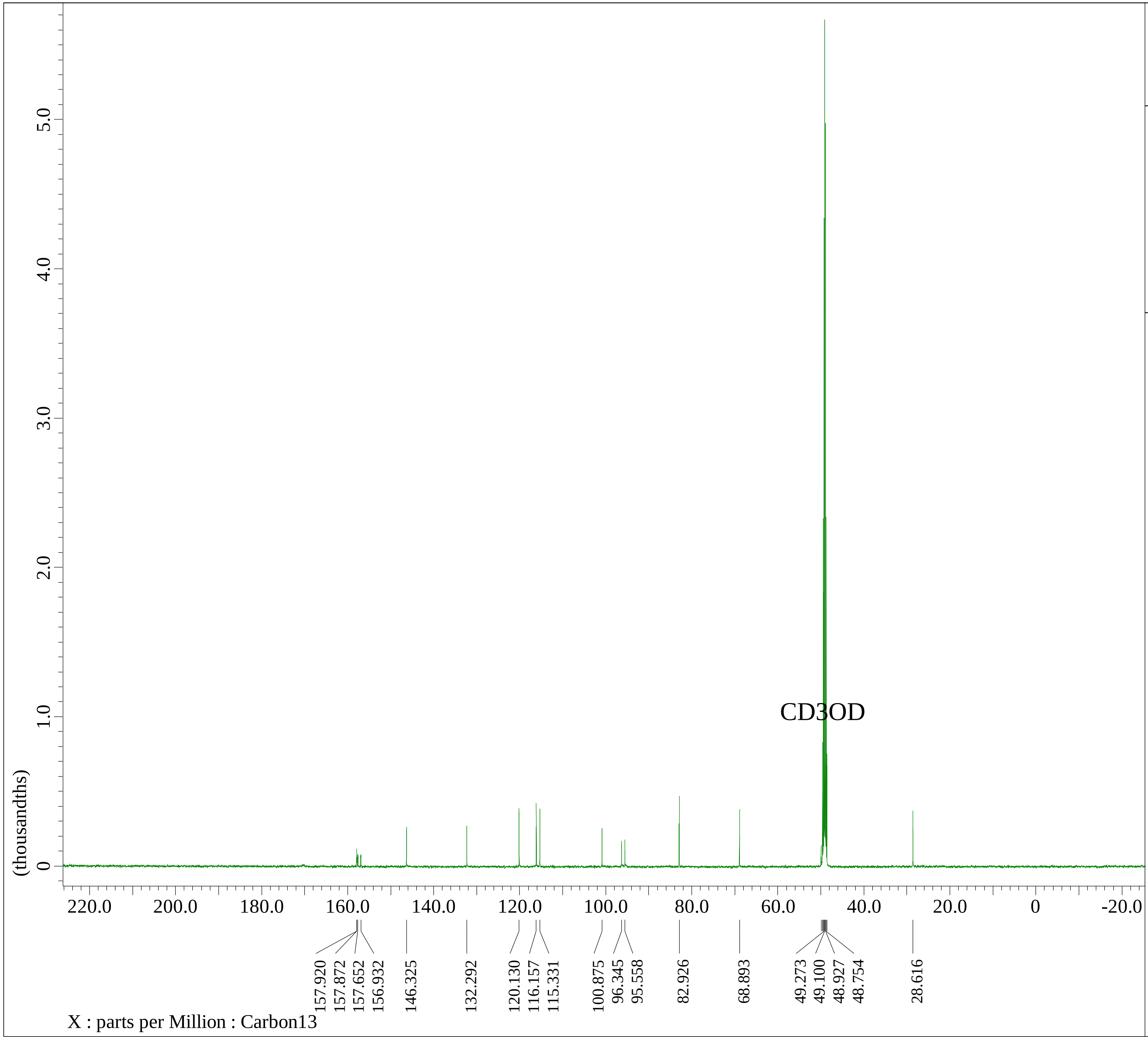

JEOL

```
---- PROCESSING PARAMETERS ----
sexp( 2.0[Hz], 0.0[s] )
trapezoid( 0[%], 0[%], 80[%], 100[%] )
zerofill( 1 )
fft( 1, TRUE, TRUE )
machinephase
ppm

Derived from: SORAYA-1_Carbon-1-1.jdf
```

```
Filename                  = SORAYA-1_Carbon-1-
Author                    = delta
Experiment                = carbon.jxp
Sample_Id                 = SORAYA-1
Solvent                   = METHANOL-D4
Actual_Start_Time         =  2-JAN-2020 09:05:
Revision_Time             =  2-JAN-2020 13:00:

Comment                   = single pulse decou
Data_Format               = 1D COMPLEX
Dim_Size                  = 26214
X_Domain                  = Carbon13
Dim_Title                 = Carbon13
Dim_Units                 = [ppm]
Dimensions                = X
Spectrometer              = JNM-ECZ500R/S1

Field_Strength            = 11.7473579[T] (500
X_Acq_Duration            = 0.82837504[s]
X_Domain                  = Carbon13
X_Freq                    = 125.76529768[MHz]
X_Offset                  = 100[ppm]
X_Points                  = 32768
X_Prescans                = 4
X_Resolution              = 1.20718268[Hz]
X_Sweep                   = 39.55696203[kHz]
X_Sweep_Clipped           = 31.64556962[kHz]
Irr_Domain                = Proton
Irr_Freq                  = 500.15991521[MHz]
Irr_Offset                = 5.0[ppm]
Blanking                  = 2[us]
Clipped                   = FALSE
Scans                     = 5000
Total_Scans               = 5000

Relaxation_Delay          = 2[s]
Recvr_Gain                = 36
Temp_Get                  = 18.4[dC]
X_90_Width                = 11.3[us]
X_Acq_Time                = 0.82837504[s]
X_Angle                   = 30[deg]
X_Atn                     = 11[dB]
X_Pulse                   = 3.76666667[us]
Irr_Atn_Dec               = 30.11[dB]
Irr_Atn_Dec_Calc          = 30.11[dB]
Irr_Atn_Dec_Default_Calc  = 30.11[dB]
Irr_Atn_Noe               = 30.11[dB]
Irr_Dec_Bandwidth_Hz      = 5.97826087[kHz]
```

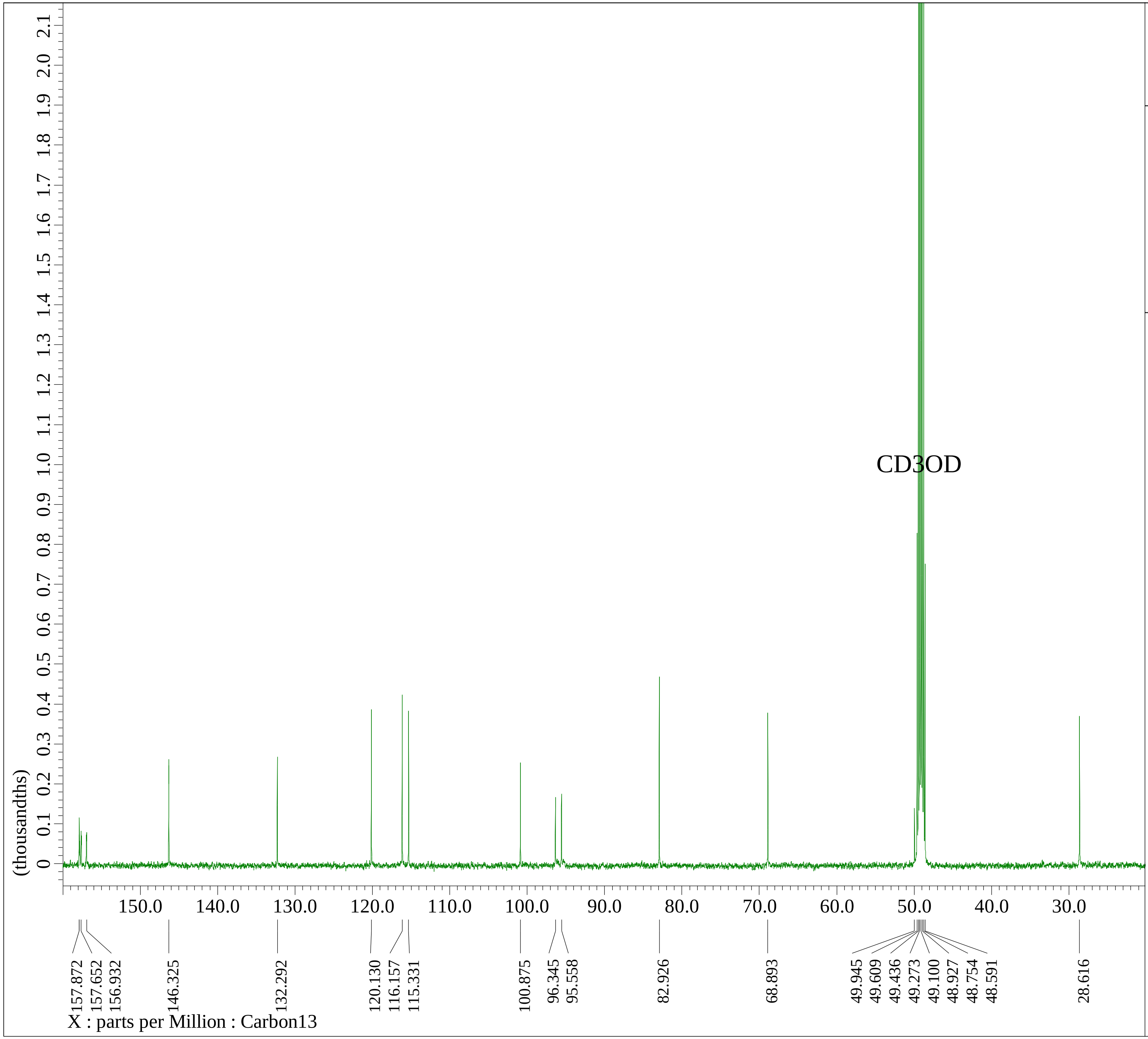


```
---- PROCESSING PARAMETERS ----
sexp( 2.0[Hz], 0.0[s] )
trapezoid( 0[%], 0[%], 80[%], 100[%] )
zerofill( 1 )
fft( 1, TRUE, TRUE )
machinephase
ppm

Derived from: SORAYA-1_Carbon-1-1.jdf
```

```
Filename                 = SORAYA-1_Carbon-1-
Author                   = delta
Experiment               = carbon.jxp
Sample_Id                = SORAYA-1
Solvent                  = METHANOL-D4
Actual_Start_Time        =  2-JAN-2020 09:05:
Revision_Time            =  2-JAN-2020 13:00:

Comment                  = single pulse decou
Data_Format              = 1D COMPLEX
Dim_Size                 = 26214
X_Domain                 = Carbon13
Dim_Title                = Carbon13
Dim_Units                = [ppm]
Dimensions               = X
Spectrometer             = JNM-ECZ500R/S1

Field_Strength           = 11.7473579[T] (500
X_Acq_Duration           = 0.82837504[s]
X_Domain                 = Carbon13
X_Freq                   = 125.76529768[MHz]
X_Offset                 = 100[ppm]
X_Points                 = 32768
X_Prescans               = 4
X_Resolution             = 1.20718268[Hz]
X_Sweep                  = 39.55696203[kHz]
X_Sweep_Clipped          = 31.64556962[kHz]
Irr_Domain               = Proton
Irr_Freq                 = 500.15991521[MHz]
Irr_Offset               = 5.0[ppm]
Blanking                 = 2[us]
Clipped                  = FALSE
Scans                    = 5000
Total_Scans              = 5000

Relaxation_Delay         = 2[s]
Recvr_Gain               = 36
Temp_Get                 = 18.4[dC]
X_90_Width               = 11.3[us]
X_Acq_Time               = 0.82837504[s]
X_Angle                  = 30[deg]
X_Atn                    = 11[dB]
X_Pulse                  = 3.76666667[us]
Irr_Atn_Dec              = 30.11[dB]
Irr_Atn_Dec_Calc         = 30.11[dB]
Irr_Atn_Dec_Default_Calc = 30.11[dB]
Irr_Atn_Noe              = 30.11[dB]
Irr_Dec_Bandwidth_Hz     = 5.97826087[kHz]
```

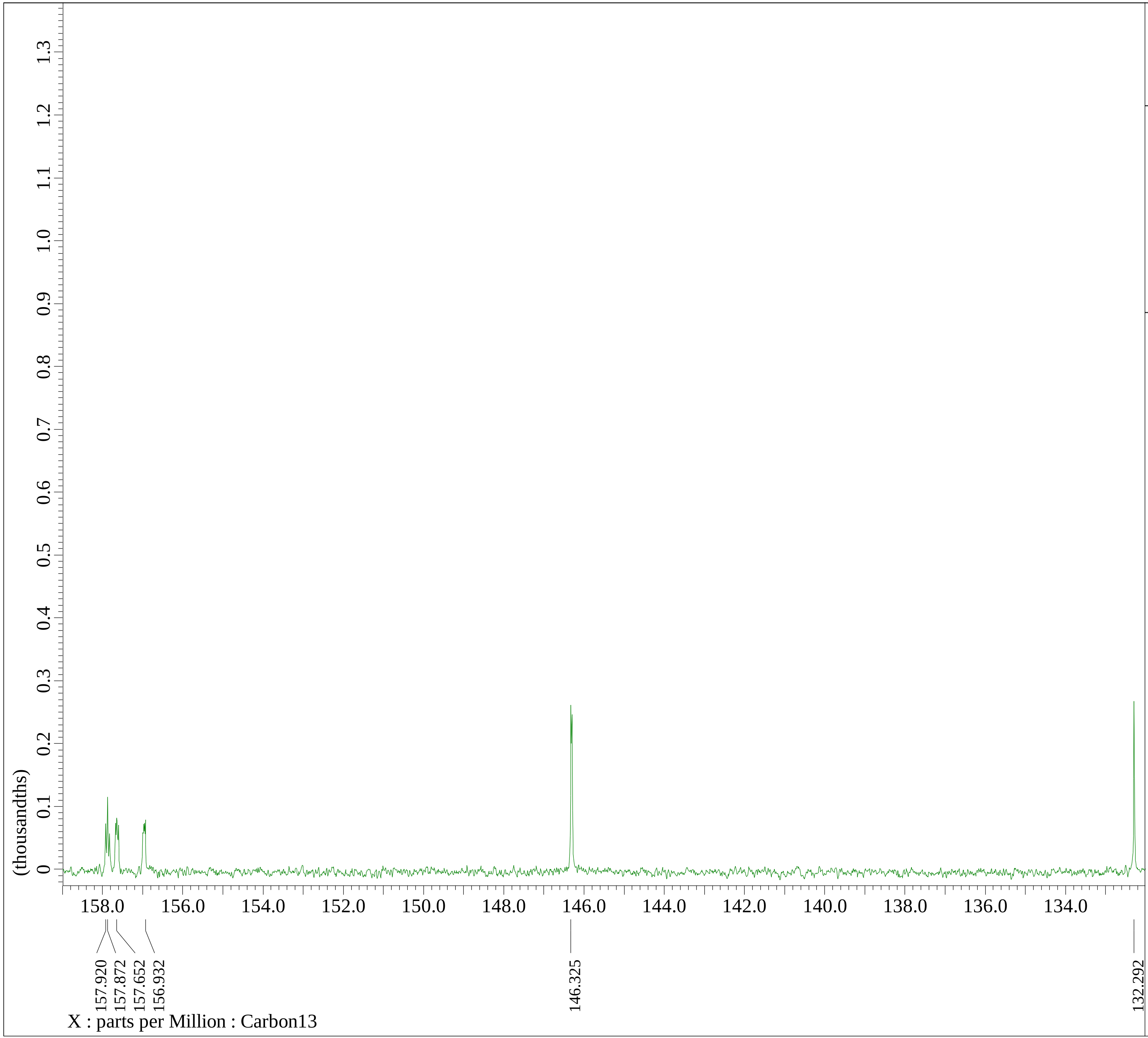

JEOL

```
---- PROCESSING PARAMETERS ----
sexp( 2.0[Hz], 0.0[s] )
trapezoid( 0[%], 0[%], 80[%], 100[%] )
zerofill( 1 )
fft( 1, TRUE, TRUE )
machinephase
ppm

Derived from: SORAYA-1_Carbon-1-1.jdf
```

```
Filename                  = SORAYA-1_Carbon-1-
Author                    = delta
Experiment                = carbon.jxp
Sample_Id                 = SORAYA-1
Solvent                   = METHANOL-D4
Actual_Start_Time         =  2-JAN-2020 09:05:
Revision_Time             =  2-JAN-2020 13:00:

Comment                   = single pulse decou
Data_Format               = 1D COMPLEX
Dim_Size                  = 26214
X_Domain                  = Carbon13
Dim_Title                 = Carbon13
Dim_Units                 = [ppm]
Dimensions                = X
Spectrometer              = JNM-ECZ500R/S1

Field_Strength            = 11.7473579[T] (500
X_Acq_Duration            = 0.82837504[s]
X_Domain                  = Carbon13
X_Freq                    = 125.76529768[MHz]
X_Offset                  = 100[ppm]
X_Points                  = 32768
X_Prescans                = 4
X_Resolution              = 1.20718268[Hz]
X_Sweep                   = 39.55696203[kHz]
X_Sweep_Clipped           = 31.64556962[kHz]
Irr_Domain                = Proton
Irr_Freq                  = 500.15991521[MHz]
Irr_Offset                = 5.0[ppm]
Blanking                  = 2[us]
Clipped                   = FALSE
Scans                     = 5000
Total_Scans               = 5000

Relaxation_Delay          = 2[s]
Recvr_Gain                = 36
Temp_Get                  = 18.4[dC]
X_90_Width                = 11.3[us]
X_Acq_Time                = 0.82837504[s]
X_Angle                   = 30[deg]
X_Atn                     = 11[dB]
X_Pulse                   = 3.76666667[us]
Irr_Atn_Dec               = 30.11[dB]
Irr_Atn_Dec_Calc          = 30.11[dB]
Irr_Atn_Dec_Default_Calc  = 30.11[dB]
Irr_Atn_Noe               = 30.11[dB]
Irr_Dec_Bandwidth_Hz      = 5.97826087[kHz]
```

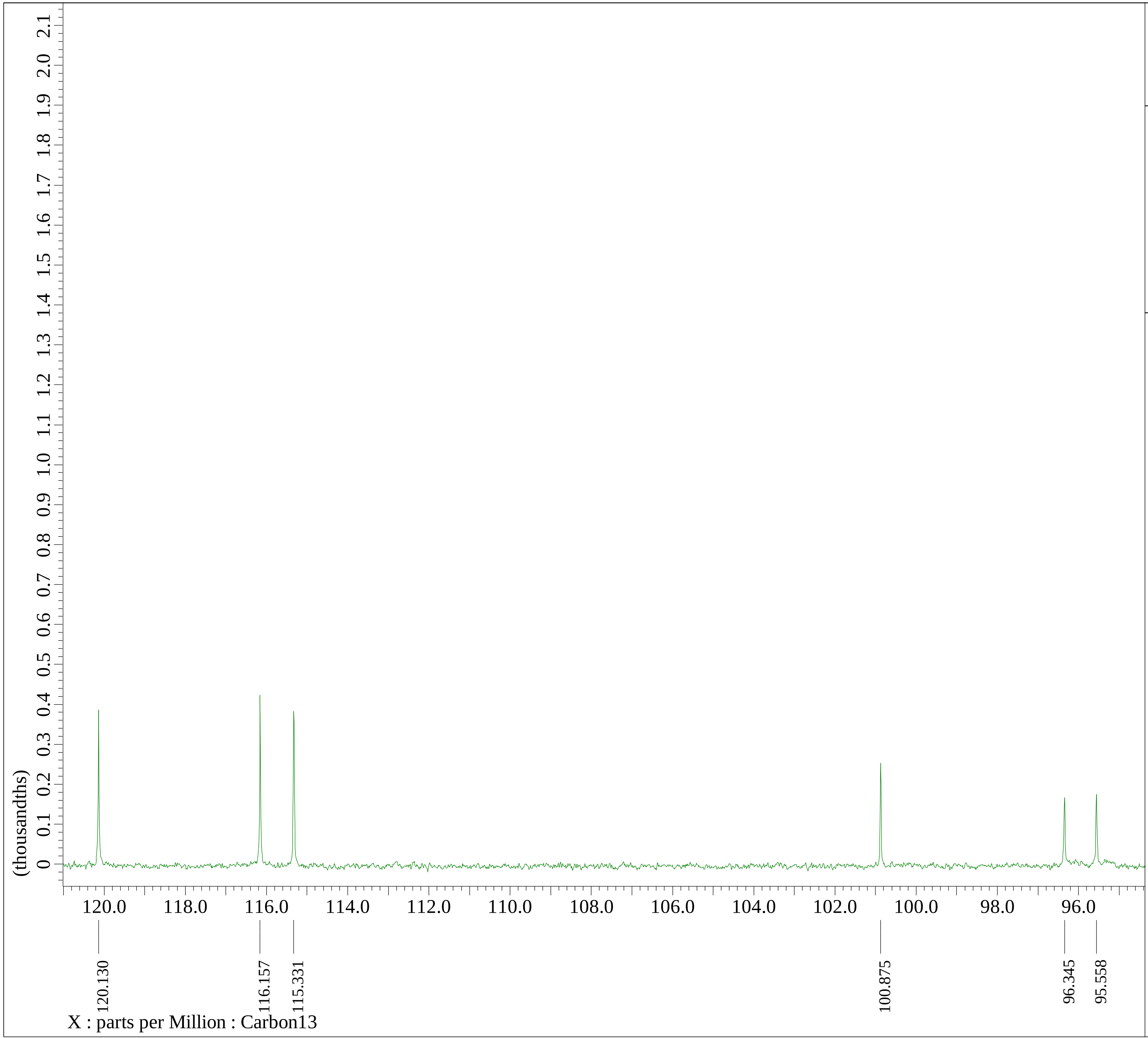


```
---- PROCESSING PARAMETERS ----
sexp( 2.0[Hz], 0.0[s] )
trapezoid( 0[%], 0[%], 80[%], 100[%] )
zerofill( 1 )
fft( 1, TRUE, TRUE )
machinephase
ppm

Derived from: SORAYA-1_Carbon-1-1.jdf
```

```
Filename                    = SORAYA-1_Carbon-1-
Author                      = delta
Experiment                  = carbon.jxp
Sample_Id                   = SORAYA-1
Solvent                     = METHANOL-D4
Actual_Start_Time           =  2-JAN-2020 09:05:
Revision_Time               =  2-JAN-2020 13:00:

Comment                     = single pulse decou
Data_Format                 = 1D COMPLEX
Dim_Size                    = 26214
X_Domain                    = Carbon13
Dim_Title                   = Carbon13
Dim_Units                   = [ppm]
Dimensions                  = X
Spectrometer                = JNM-ECZ500R/S1

Field_Strength              = 11.7473579[T] (500
X_Acq_Duration              = 0.82837504[s]
X_Domain                    = Carbon13
X_Freq                      = 125.76529768[MHz]
X_Offset                    = 100[ppm]
X_Points                    = 32768
X_Prescans                  = 4
X_Resolution                = 1.20718268[Hz]
X_Sweep                     = 39.55696203[kHz]
X_Sweep_Clipped             = 31.64556962[kHz]
Irr_Domain                  = Proton
Irr_Freq                    = 500.15991521[MHz]
Irr_Offset                  = 5.0[ppm]
Blanking                    = 2[us]
Clipped                     = FALSE
Scans                       = 5000
Total_Scans                 = 5000

Relaxation_Delay            = 2[s]
Recvr_Gain                  = 36
Temp_Get                    = 18.4[dC]
X_90_Width                  = 11.3[us]
X_Acq_Time                  = 0.82837504[s]
X_Angle                     = 30[deg]
X_Atn                       = 11[dB]
X_Pulse                     = 3.76666667[us]
Irr_Atn_Dec                 = 30.11[dB]
Irr_Atn_Dec_Calc            = 30.11[dB]
Irr_Atn_Dec_Default_Calc    = 30.11[dB]
Irr_Atn_Noe                 = 30.11[dB]
Irr_Dec_Bandwidth_Hz        = 5.97826087[kHz]
```
