# Supplementary material for: Nanoparticulate System for the Transdermal Delivery of Catechin as an Antihypercholesterol: In Vitro and In Vivo Evaluations
Source: Pharmaceuticals (Basel). 2022 Sep 13;15(9):1142. doi: 10.3390/ph15091142 (PMC9505170; doi:10.3390/ph15091142)
Supplement: Supplementary file 1 [file pharmaceuticals-15-01142-s001.zip › Figure S9. PSA PDI Zeta Potential of Transethosome Catechin.pdf]

F2 Katekin 1%_4179.nsz

Measurement Results

Date	:	Senin, 01 Februari 2021 14.03.07
Measurement Type	:	Particle Size
Sample Name	:	F2 Katekin 1%
Scattering Angle	:	90
Temperature of the Holder	:	25.0 deg. C
Dispersion Medium Viscosity	:	0.896 mPa.s
Transmission Intensity before Meas.	:	28320
Measurement Gate Time	:	---
Distribution Form	:	|Standard|
Distribution Form(Dispersity)	:	Polydisperse
Representation of Result	:	Scattering Light Intensity
Count Rate	:	1382 kCPS

Calculation Results

Peak No.	S.P.Area Ratio	Mean	S. D.	Mode
1	1.00	177.2 nm	62.7 nm	143.0 nm
2	---	--- nm	--- nm	--- nm
3	---	--- nm	--- nm	--- nm
Total	1.00	177.2 nm	62.7 nm	143.0 nm

Histogram Operations

Size (Median)	:	162.3 nm
% Cumulative (2)	:	10.0 (%) - 110.4 (nm)
% Cumulative (4)	:	30.0 (%) - 135.8 (nm)
% Cumulative (6)	:	50.0 (%) - 162.3 (nm)
% Cumulative (8)	:	70.0 (%) - 198.3 (nm)
Mean	:	177.2 nm

Cumulant Operations

Z-Average	:	163.6 nm
PI	:	0.239

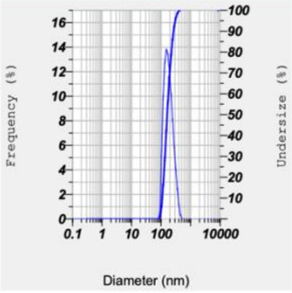

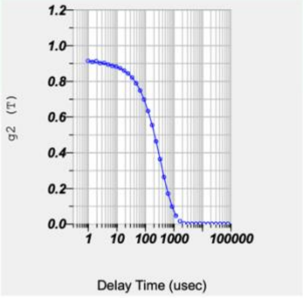

F2 Katekin 1%_4284.nsz

Measurement Results

Date	:	Selasa, 09 Februari 2021 11.51.53
Measurement Type	:	Particle Size
Sample Name	:	F2 Katekin 1%
Scattering Angle	:	90
Temperature of the Holder	:	25.0 deg. C
Dispersion Medium Viscosity	:	0.895 mPa.s
Transmission Intensity before Meas.	:	28283
Measurement Gate Time	:	---
Distribution Form	:	|Standard|
Distribution Form(Dispersity)	:	Monodisperse
Representation of Result	:	Scattering Light Intensity
Count Rate	:	1701 kCPS

Calculation Results

Peak No.	S.P.Area Ratio	Mean	S. D.	Mode
1	1.00	165.3 nm	39.5 nm	160.5 nm
2	---	--- nm	--- nm	--- nm
3	---	--- nm	--- nm	--- nm
Total	1.00	165.3 nm	39.5 nm	160.5 nm

Histogram Operations

Size (Median)	:	159.8 nm
% Cumulative (2)	:	10.0 (%) - 118.0 (nm)
% Cumulative (4)	:	30.0 (%) - 139.4 (nm)
% Cumulative (6)	:	50.0 (%) - 159.8 (nm)
% Cumulative (8)	:	70.0 (%) - 184.0 (nm)
Mean	:	165.3 nm

Cumulant Operations

Z-Average	:	166.5 nm
PI	:	0.025

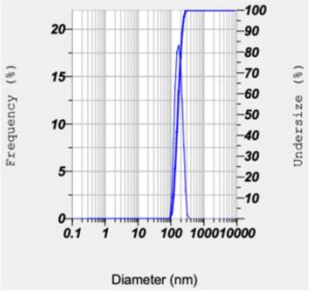

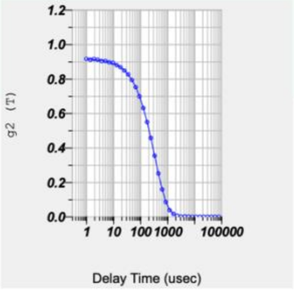

Transetosom Katekin 1% _4487.nsz

Measurement Results

Date	:	Senin, 22 Februari 2021 15.24.36
Measurement Type	:	Particle Size
Sample Name	:	Transetosom Katekin 1%
Scattering Angle	:	90
Temperature of the Holder	:	25.0 deg. C
Dispersion Medium Viscosity	:	0.895 mPa.s
Transmission Intensity before Meas.	:	27646
Measurement Gate Time	:	---
Distribution Form	:	|Standard|
Distribution Form(Dispersity)	:	Polydisperse
Representation of Result	:	Scattering Light Intensity
Count Rate	:	1527 kCPS

Calculation Results

Peak No.	S.P.Area Ratio	Mean	S. D.	Mode
1	1.00	185.8 nm	70.3 nm	160.6 nm
2	---	--- nm	--- nm	--- nm
3	---	--- nm	--- nm	--- nm
Total	1.00	185.8 nm	70.3 nm	160.6 nm

Histogram Operations

Size (Median)	:	169.3 nm
% Cumulative (2)	:	10.0 (%) - 109.8 (nm)
% Cumulative (4)	:	30.0 (%) - 138.7 (nm)
% Cumulative (6)	:	50.0 (%) - 169.3 (nm)
% Cumulative (8)	:	70.0 (%) - 211.1 (nm)
Mean	:	185.8 nm

Cumulant Operations

Z-Average	:	166.2 nm
PI	:	0.076

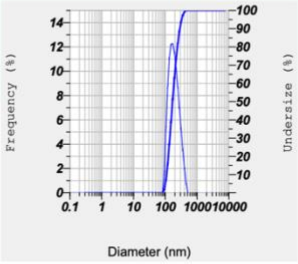

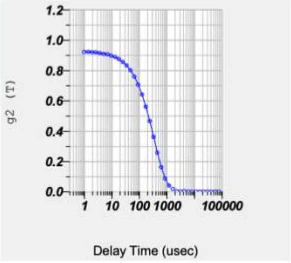

PSA and PDI of Catechin Transethisime 1%

F2 Katekin 1%_4191.nzt

Measurement Results

Date	:	Senin, 01 Februari 2021 15.56.41
Measurement Type	:	Zeta Potential
Sample Name	:	F2 Katekin 1%
Dispersion Medium Information	:	
Temperature of the Holder	:	24.9 deg. C
Dispersion Medium Viscosity	:	0.897 mPa.s
Conductivity	:	0.192 mS/cm
Electrode Voltage	:	3.3 V

Calculation Results

Peak No.	Zeta Potential	Electrophoretic Mobility
1	-8.8 mV	-0.000068 cm2/Vs
2	--- mV	--- cm2/Vs
3	--- mV	--- cm2/Vs

Zeta Potential (Mean) : -8.8 mV
Electrophoretic Mobility Mean : -0.000068 cm^2/Vs

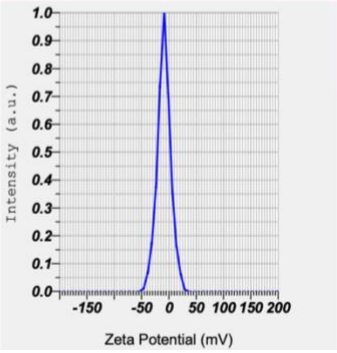

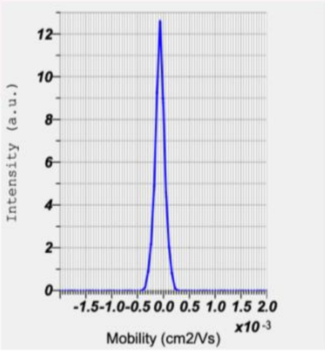

Transetosom Katekin 1%_4496.nzt

Measurement Results

Date	:	Senin, 22 Februari 2021 16.28.15
Measurement Type	:	Zeta Potential
Sample Name	:	Transetosom Katekin 1%
Dispersion Medium Information	:	
Temperature of the Holder	:	25.0 deg. C
Dispersion Medium Viscosity	:	0.895 mPa.s
Conductivity	:	0.197 mS/cm
Electrode Voltage	:	3.3 V

Calculation Results

Peak No.	Zeta Potential	Electrophoretic Mobility
1	-8.3 mV	-0.000064 cm2/Vs
2	--- mV	--- cm2/Vs
3	--- mV	--- cm2/Vs

Zeta Potential (Mean) : -8.3 mV
Electrophoretic Mobility Mean : -0.000064 cm^2/Vs

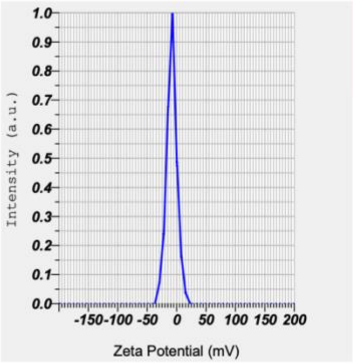

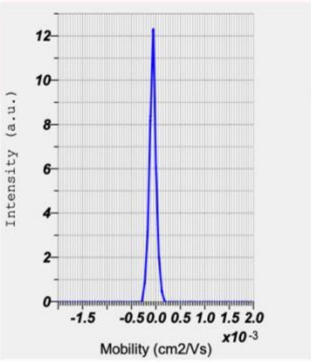

F2 Katekin 1%_4308.nzt

Measurement Results

Date	:	Selasa, 09 Februari 2021 14.12.16
Measurement Type	:	Zeta Potential
Sample Name	:	F2 Katekin 1%
Dispersion Medium Information	:	
Temperature of the Holder	:	25.0 deg. C
Dispersion Medium Viscosity	:	0.895 mPa.s
Conductivity	:	0.193 mS/cm
Electrode Voltage	:	3.3 V

Calculation Results

Peak No.	Zeta Potential	Electrophoretic Mobility
1	-17.7 mV	-0.000137 cm2/Vs
2	--- mV	--- cm2/Vs
3	--- mV	--- cm2/Vs

Zeta Potential (Mean) : -17.7 mV
Electrophoretic Mobility Mean : -0.000137 cm^2/Vs

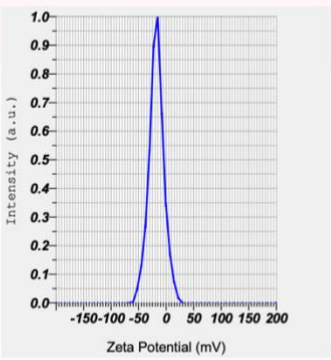

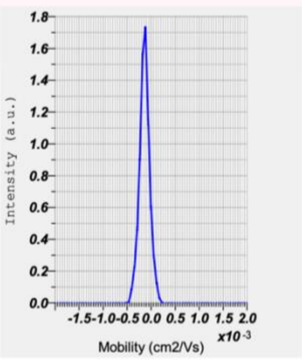

Zeta Potential of Catechin Transethosome 1%
