# Supplementary material for: Nanoparticulate System for the Transdermal Delivery of Catechin as an Antihypercholesterol: In Vitro and In Vivo Evaluations
Source: Pharmaceuticals (Basel). 2022 Sep 13;15(9):1142. doi: 10.3390/ph15091142 (PMC9505170; doi:10.3390/ph15091142)
Supplement: Supplementary file 1 [file pharmaceuticals-15-01142-s001.zip › Figure S5. Spectrum IR Transethosomal Catechin.pdf]

thermo scientific

transetosome-katekin 0,5 2/11/2021 9:28:16 AM

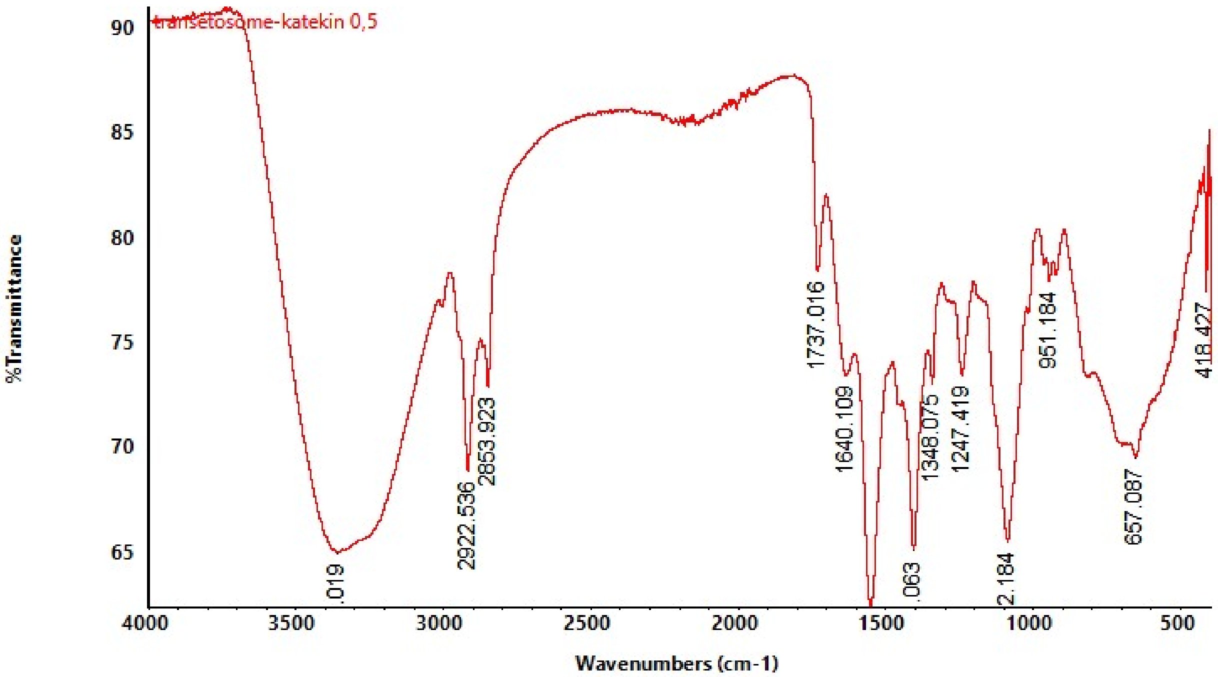

Title: transetosome-katekin 0,5
Number of sample scans: 16
Number of background scans: 16
Instrument Serial: BDM1910155
Smart Accy: V67654
Model: Summit
Source: IR
Detector: DTGS KBr
Smart Accessory Title: Everest ATR
Smart Accessory ID: V67654
Crystal type: : Diamond
Beamsplitter: KBr
Sample spacing: 1.0
Digitizer bits: 24
Optical velocity: 0.4747
Aperture: 100.0
Sample gain: 1.0
High pass filter: 1.0
Low pass filter: 11000.0
Comments: None
