# Supplementary figures and images for: Nanoparticulate System for the Transdermal Delivery of Catechin as an Antihypercholesterol: In Vitro and In Vivo Evaluations
Source: Pharmaceuticals (Basel). 2022 Sep 13;15(9):1142. doi: 10.3390/ph15091142 (PMC9505170; doi:10.3390/ph15091142)

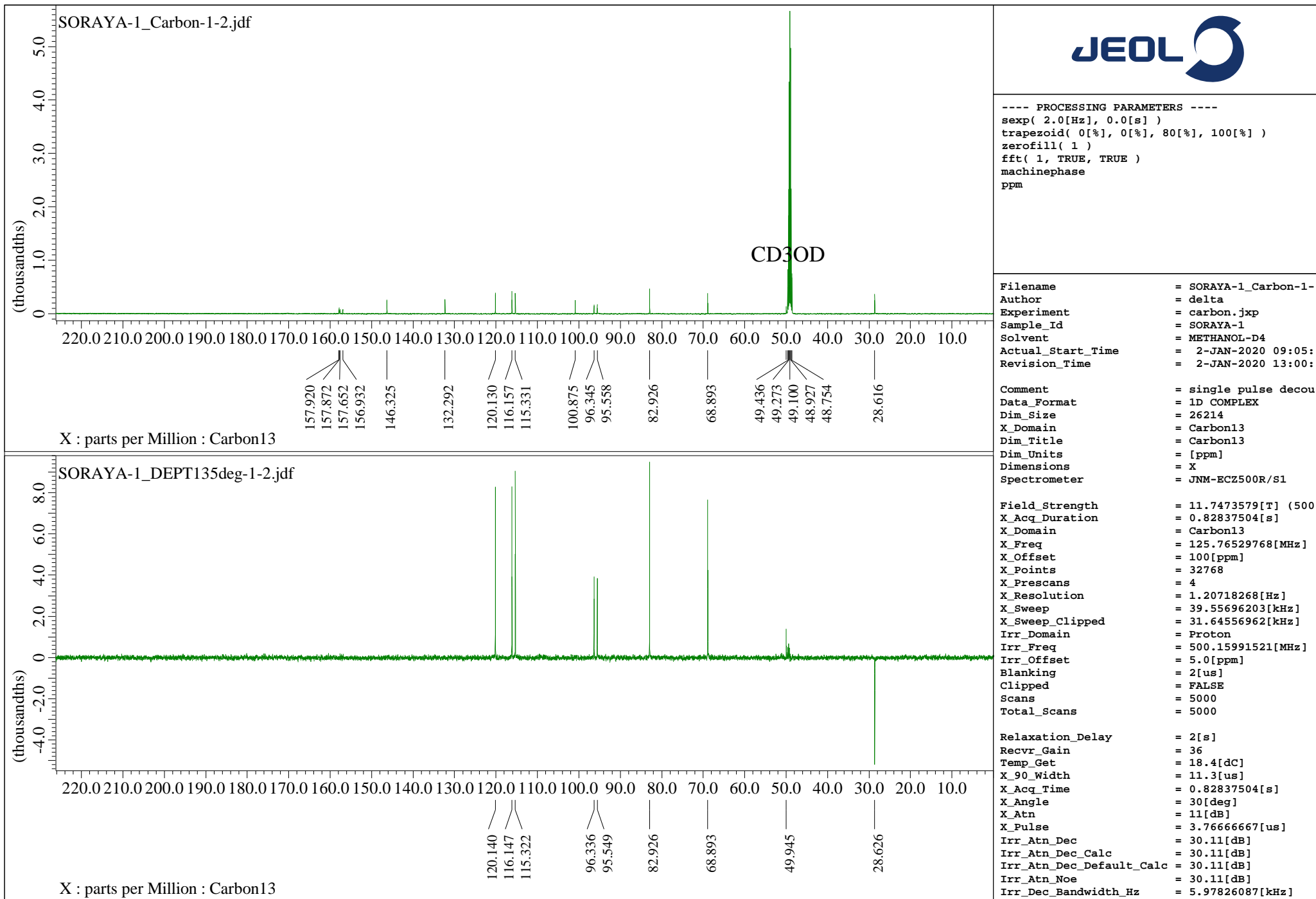

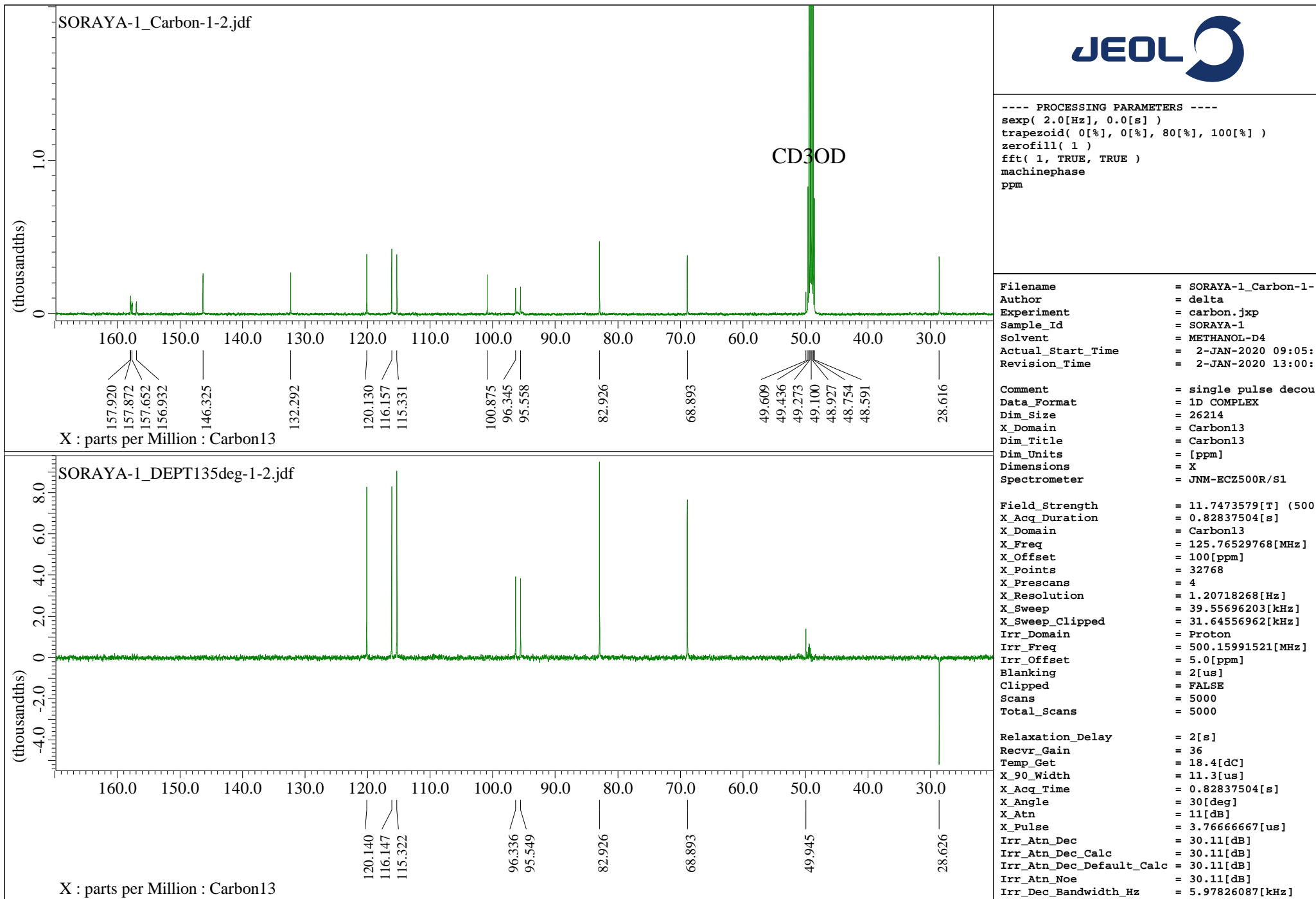

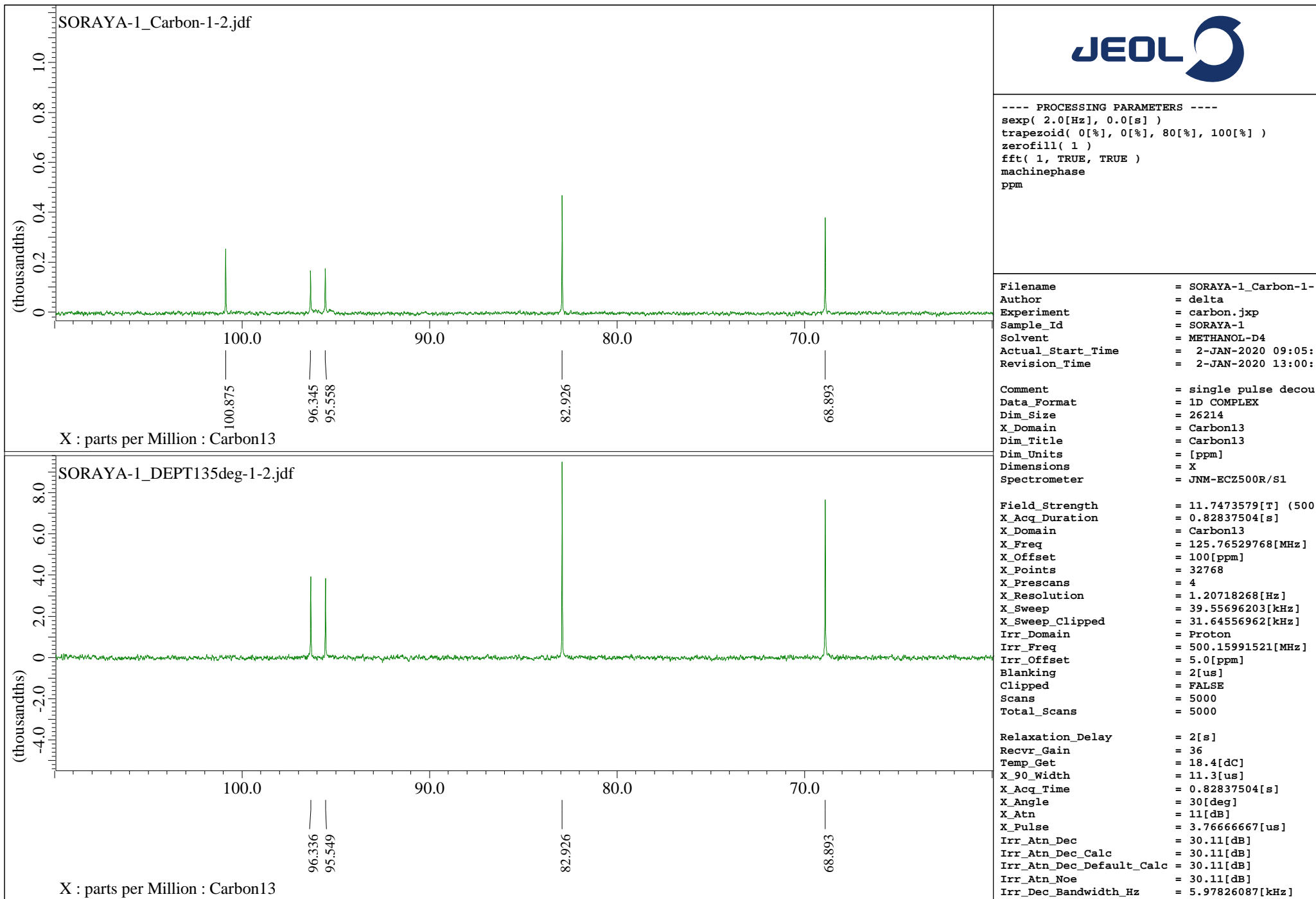

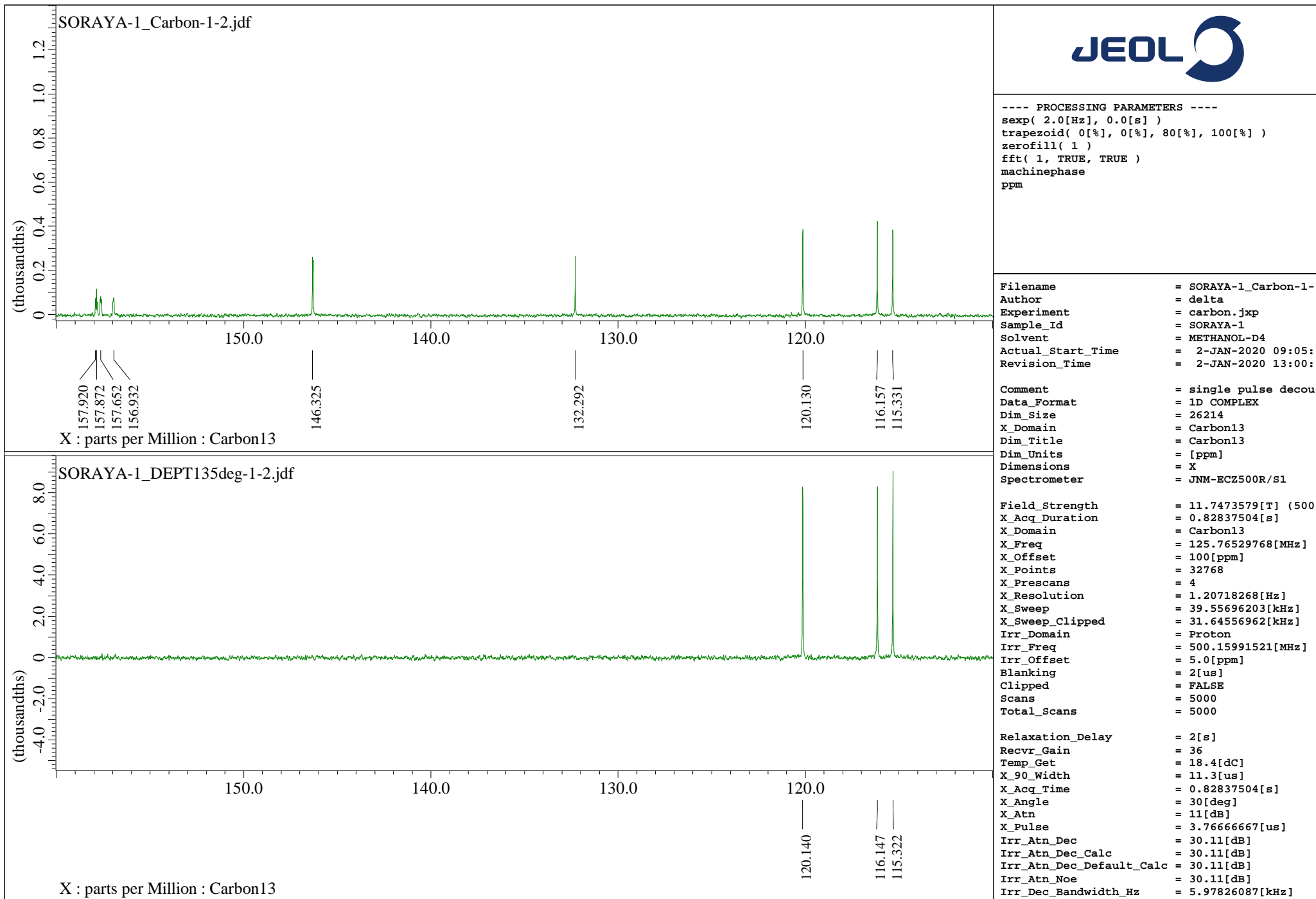

Supplement: Supplementary file 1 [file pharmaceuticals-15-01142-s001.zip › Figure S8. NMR Overlay Spectrum.pdf]

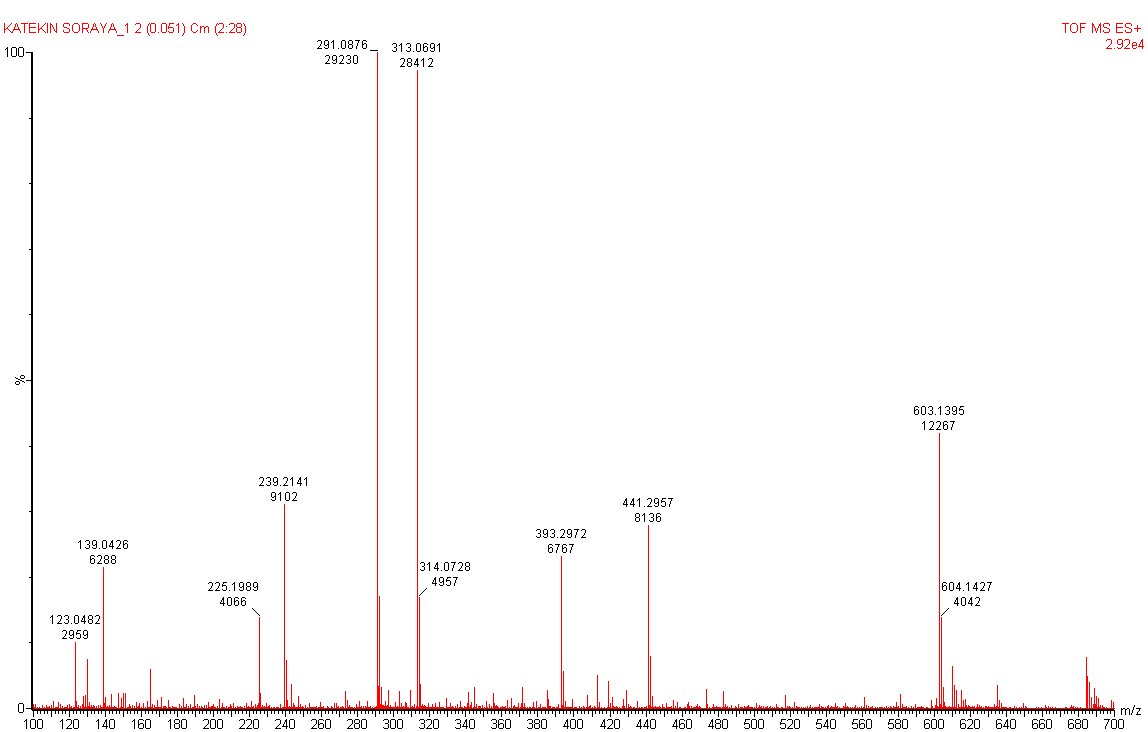

Supplement: Supplementary file 1 [file pharmaceuticals-15-01142-s001.zip › Figure S2. MS Spectrum.bmp]

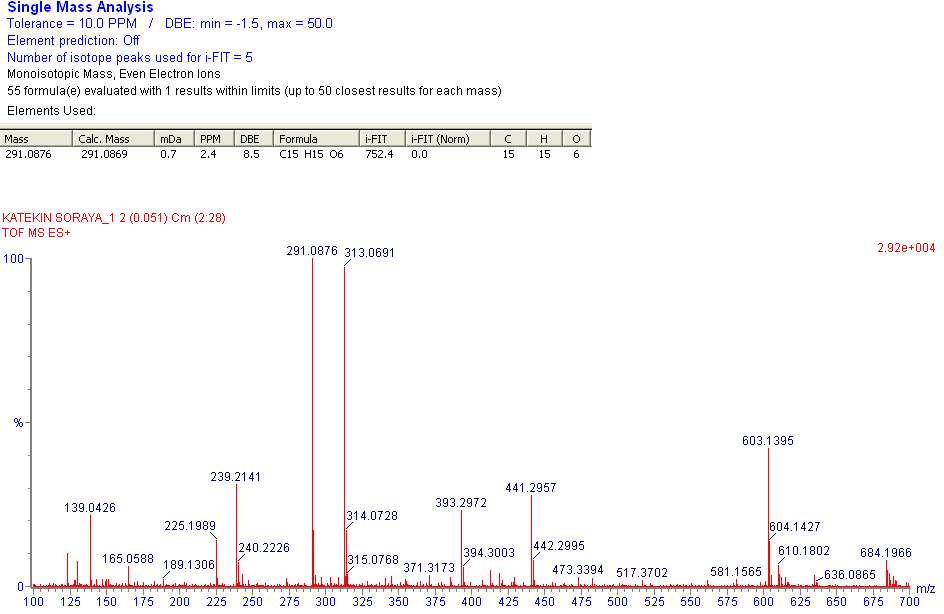

Supplement: Supplementary file 1 [file pharmaceuticals-15-01142-s001.zip › Figure S1. MS Spectrum Prediction.bmp]
